# Supramolecular Stability of Benzene-1,3,5-tricarboxamide Supramolecular Polymers in Biological Media: Beyond the Stability–Responsiveness Trade-off

**DOI:** 10.1021/jacs.2c08528

**Published:** 2022-11-11

**Authors:** Edgar Fuentes, Yeray Gabaldón, Mario Collado, Shikha Dhiman, José Augusto Berrocal, Silvia Pujals, Lorenzo Albertazzi

**Affiliations:** † Department of Biological Chemistry, Institute for Advanced Chemistry of Catalonia (IQAC-CSIC), Jordi Girona 18-26, 08034 Barcelona, Spain; ‡ Institute for Bioengineering of Catalonia, The Barcelona Institute of Science and Technology, Baldiri Reixac 15-21, 08028 Barcelona, Spain; § Laboratory of Macromolecular and Organic Chemistry, Eindhoven University of Technology, P. O. Box 513, 5600 MB Eindhoven, The Netherlands; ∥ Institute of Complex Molecular Systems, Eindhoven University of Technology, P. O. Box 513, 5600 MB Eindhoven, The Netherlands; ⊥ Adolphe Merkle Institute, University of Fribourg, Chemin des Verdiers 4, 1700 Fribourg, Switzerland; # Department of Biomedical Engineering, Eindhoven University of Technology, P. O. Box 513, 5612 AZ Eindhoven, The Netherlands

## Abstract

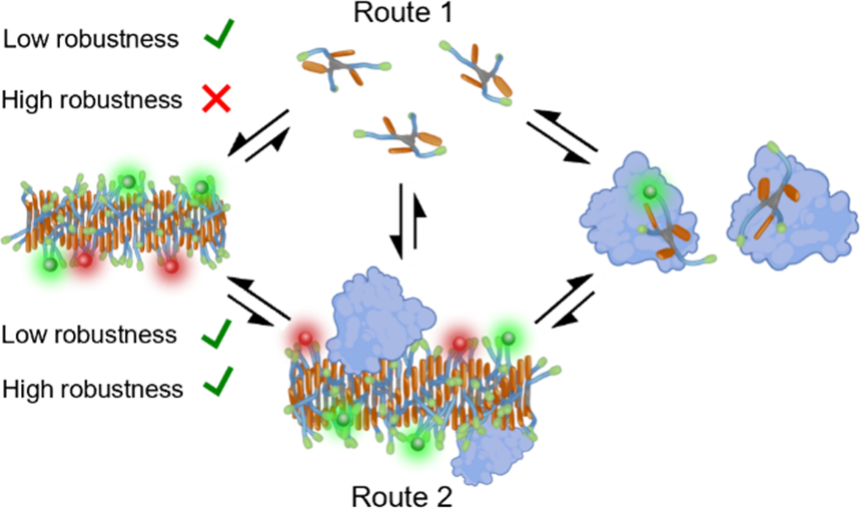

Supramolecular assemblies have been gaining attention in recent years in the field of drug delivery because of their unique formulation possibilities and adaptive behavior. Their non-covalent nature allows for their self-assembly formulation and responsiveness to stimuli, an appealing feature to trigger a therapeutic action with spatiotemporal control. However, facing in vivo conditions is very challenging for non-covalent structures. Dilution and proteins in blood can have a direct impact on self-assembly, destabilizing the supramolecules and leading to a premature and uncontrolled cargo release. To rationalize this behavior, we designed three monomers exhibiting distinct hydrophobic cores that self-assemble into photo-responsive fibers. We estimated their stability–responsiveness trade-off in vitro, finding two well-separated regimes. These are low-robustness regime, in which the system equilibrates quickly and responds readily to stimuli, and high-robustness regime, in which the system equilibrates slowly and is quite insensitive to stimuli. We probed the performance of both regimes in a complex environment using Förster resonance energy transfer (FRET). Interestingly, the stability–responsiveness trade-off defines perfectly the extent of disassembly caused by dilution but not the one caused by protein interaction. This identifies a disconnection between intrinsic supramolecular robustness and supramolecular stability in the biological environment, strongly influenced by the disassembly pathway upon protein interaction. These findings shed light on the key features to address for supramolecular stability in the biological environment.

## 1 Introduction

Supramolecular polymers are present in nature as robust yet adaptive structures. As an example, cytoskeletal fibers provide the cell with robust mechanical actuators and active transport and movement, while being highly dynamic structures. Their continuous build-and-destroy dynamics allows for a fast (de)polymerization on demand, necessary for the correct cell function.^1−4^

These natural polymers have inspired chemists to create fascinating supramolecular constructions in water.^5,6^ The adaptive nature of supramolecular materials allowed to shape their response to stimuli and control their actuation in complex scenarios.^7^ Stupp and co-workers remarkably designed a fibrous supramolecular network based on peptide amphiphiles, able to reversibly switch between distinct hierarchical architectures.^8^ Meijer and co-workers showed spatiotemporal control on supramolecular polymers using ssDNA to cluster reversibly specific monomers.^9^ The groups of Gianneschi, Amir, and Thayumanavan independently reported interesting stimulus-responsive micelles, using light, pH, or biomolecules to modulate their response.^10−13^ Recently, Choi et al. have shown polymeric micelles responsive to light and enzymes, obtaining dual control over the assemblies.^14^ Besenius et al.’s lab showed supramolecular polymers responsive to pH and reactive oxygen species, used together with temperature to control the hydrogelation and properties of the material.^15^

Despite these remarkable advances, there are still many challenges to tackle before the final application. One of these challenges revolves around the supramolecular integrity in complex environments, where multiple biomolecular interactions can impact the assembled structure.

In the nanomedicine world, in which the temperature window is very narrow, the supramolecular destabilization usually comes from the decrease in the free monomer availability (sequestering, dilution, degradation). This effect is alleviated when using supramolecular bulk materials (e.g., hydrogels), given the huge concentration of monomer, the limited diffusion within, and the low surface/volume ratio.^16^ However, other applications specifically require the use of discrete structures.

Targeted drug delivery aims to use nanostructures to carry drugs through the bloodstream and deliver them selectively at the destination. This means facing massive dilution along with a variety of serum proteins and potential side interactions.^17^ These circumstances are a special challenge for self-assembled materials because they contribute to the decrease in monomer availability, promoting carrier disruption before reaching the target. Supramolecular drug delivery systems require at the same time high robustness to resist the journey and the ability to respond to stimuli to deliver the drug.

Even though it is a crucial issue, this destabilization is not very well understood. A thorough understanding about this phenomenon could lead to improved supramolecules for drug delivery. Few pioneering examples can be found in literature. Together with the group of Roey Amir, our group thoroughly studied the integrity of enzyme-responsive micelles when facing a biological environment or even biological barriers.^18,19^ These works show how the presence of proteins impacts differently depending on the stability–responsiveness balance. Furthermore, it was proven that extravasation and even the internalization by cells are affected by supramolecular stability.

Interestingly, a recent article of Pavan et al. demonstrated the interaction of specific benzene-1,3,5-tricarboxamide (BTA) fibers and monomers with serum proteins.^20^ These results suggest that BTA-stacked systems could be destabilized when used as biomaterials. There is a need for rationalizing the factors influencing supramolecular stability in complex media. This could allow to improve the designs and push forward the field.

It is known that intrinsic supramolecular stability is related to the critical aggregation concentration (CAC) values; the lower the CAC, the higher the intrinsic stability.^21−23^ However, the CAC of supramolecular polymers is often very challenging to obtain. For this reason, and as stability and responsiveness are inversely related, we believe a complete view of the stability of supramolecular polymers must evaluate the responsive abilities as well. The stability–responsiveness trade-off would define better the intrinsic robustness of the system.

In this work, we have interrogated the stability/photo-responsive abilities of BTA monomers with different hydrophobic/hydrophilic ratios. Studying the stability–responsiveness trade-off unveiled two well-separated regimes in vitro, one with low stability and high responsivity and a second one of high stability and low responsivity. Relating these regimes with the behavior upon a hypothetical intravenous injection is of great significance. Förster resonance energy transfer is a powerful quantitative tool^24^ that allowed us to monitor the assembly state of the supramolecular polymers in a complex environment. Remarkably, response to dilution matches perfectly with the two stability/responsiveness regimes, the high stability/low responsiveness being translated into high resistance to dilution. However, when proteins were present, the difference between the two regimes blurred, implying that supramolecular stability in the biological environment goes beyond a high stability/responsiveness balance. These results give insights on the key parameters to be optimized to achieve complete supramolecular stability in the biological environment.

## 2 Results and Discussion

### 2.1 Molecular Design and Synthesis

**Figure 1:**
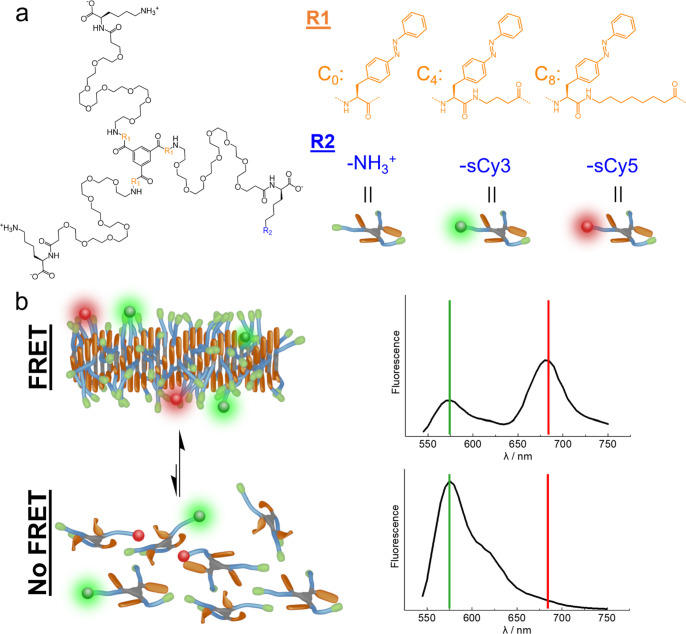
Molecular structure of C0, C4, and C8 monomers (a). Cartoon representing the FRET monitoring of self-assembly (b). Green and red lines were added to illustrate the peaks used to monitor the assembly percentage. An increase in green signal and a decrease in red signal are translated into fiber disassembly and FRET loss.

In our earlier work, we redesigned previous BTA-based systems^25,26^ introducing an azobenzene group (C0), achieving a highly responsive polymer to multiple stimuli in water.^27^ The design consisted of the C_3_-symmetric BTA as a core, bearing three identical peptide-like amphiphilic wedges. The inner hydrophobic part is constituted by an azobenzene amino acid, which is a well-known photo-responsive moiety,^28^ that demonstrated its ability to disrupt monomer stacking upon isomerization from *E* (planar) to *Z* (non-planar). This part is followed by an octaethylene glycol (OEG) that ended in a C-terminal lysine. Parting from this molecule, we have designed two new monomers in search of a change in stability/responsiveness, trying to maintain as many common features as possible. To do so, and considering that hydrophobicity is essential for BTA self-assembly,^26,29^ we extended the hydrophobic core with a 4-aminobutanoic acid or an 8-aminooctanoic acid (creating, respectively, C4 and C8 monomers. Figure 1a). This strategy allows to use the same synthetic route for the three monomers. It consisted of the growth of the bearing wedges first by solid phase synthesis, from C-terminal to N-terminal using Fmoc-protected building blocks. Once the wedge is synthesized, it is coupled to the core in solution, in a convergent fashion.

Altogether, the three molecules should allow to determine the relationship between stability and responsiveness in aqueous stacked systems and relate it with their performance in the biological environment. This is possible because the different hydrophobic/hydrophilic balances lead to changes in supramolecular stability. At the same time, it affects equally the responsive capabilities, given the trade-off relation between supramolecular stability and responsiveness, in which increasing one decreases the other. How this defined trade-off for each molecule affects stability in a biological environment is of high value. The newly designed monomers allow to keep the key common features required for this study: ability to self-assemble, responsiveness to light, and the same surface identity.

To monitor the self-assembly, each molecule was also post-functionalized at the R2 position with a single sulfo-Cyanine3 (sCy3) or sulfo-Cyanine5 (sCy5), a well-studied FRET pair of dyes.^24,30,31^ This FRET pair operates in the range of 550–750 nm, well separated from the azobenzene absorption window (250–500 nm) to avoid any kind of crosstalk. Pristine monomers can be mixed with sCy3- and sCy5-labeled monomers in DMSO, achieving a well-mixed monomerically dissolved solution (see Methods). Next, we inject the solution in phosphate-buffered saline (PBS) at 25 μM in order to trigger the self-assembly into fibers. The total amount of labeled monomers, when needed, was kept at 10 mol % for an optimal FRET signal. In Figure 1b, we can observe schematically how we can use FRET to assess stability. Only assembled monomers produce a powerful FRET signal, while upon disassembly, monomers are physically separated and FRET is lost. This allows monitoring the self-assembly process.^9,32^

### 2.2 Assembly Characterization

**Figure 2:**
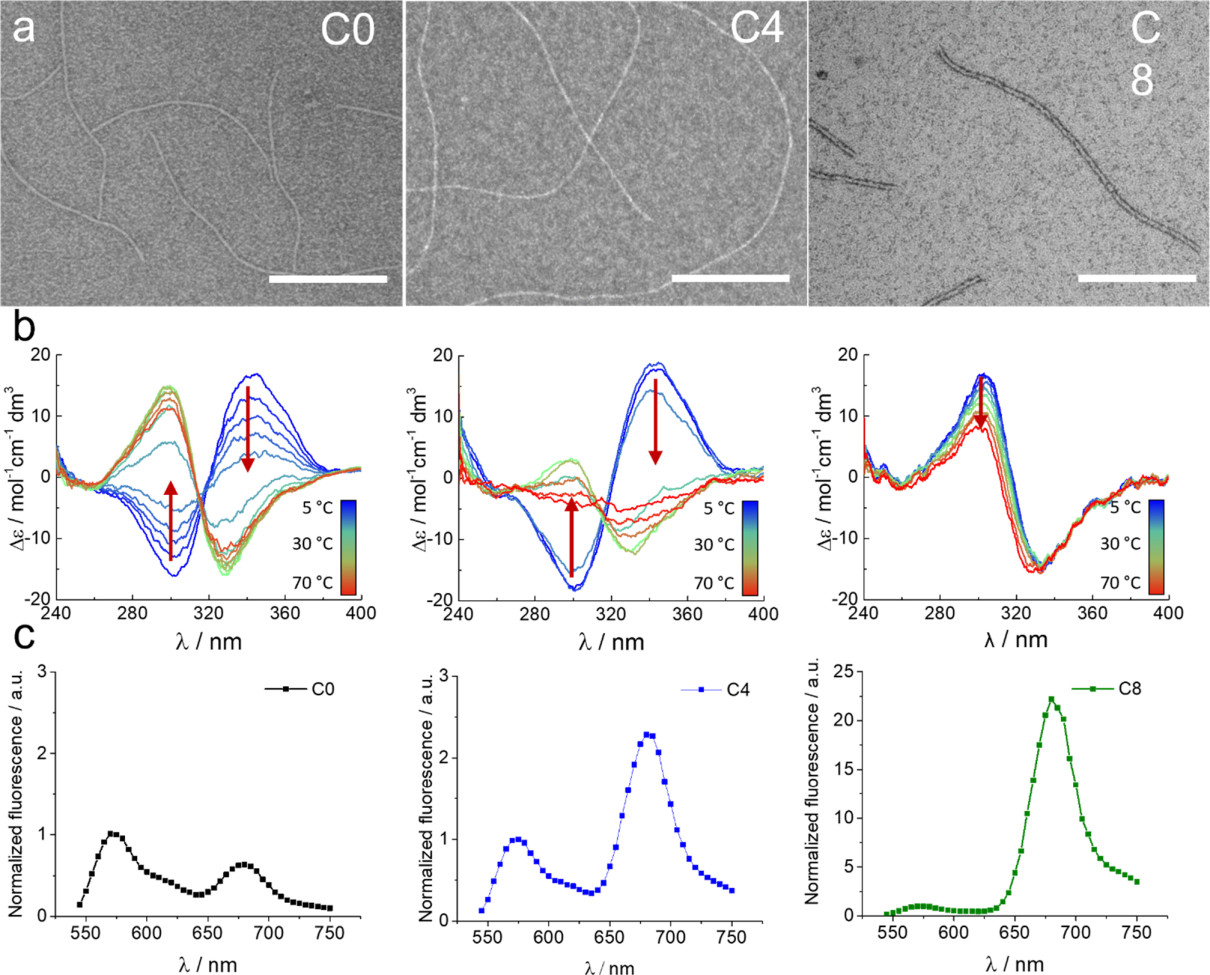
(a) TEM images of C0, C4, and C8 (from left to right). Scale bar: 200 nm. (b) CD spectra vs T of C0, C4, and C8 (from left to right), at 25 μM. (c) Fluorescence spectra of C0, C4, and C8 (from left to right), at 25 μM, 10% total labeling, and 37 °C. The FRET ratio (acceptor fluorescence/donor fluorescence) is 0.5 for C0, 2.5 for C4, and 22 for C8 (line was added to guide the eye).

First, the self-assembly was studied using transmission electron microscopy (TEM), circular dichroism (CD), and FRET (Figure 2).

TEM showed fibrillar aggregates for the three monomers. Interestingly, C0 and C4 present a similar morphology, apparently single fibers of ∼6.5 nm in width but different lengths, ∼300 nm and a few micrometers, respectively (Figure 2a). Instead, monomer C8 seems to self-assemble more into tight bundles of ∼300 nm in length and ∼8.3 nm in width. Increasing hydrophobicity of C0 to C4 increased the length of the fibers. However, increasing hydrophobicity from C4 to C8 resulted in shorter fibers and apparent changes in thickness/bundling. This is an interesting effect given that one could expect an increase in length when increasing hydrophobic forces, but only if the internal structure of the fiber is the same. In this case, we observed an inversed CD signature for C8 and increased thickness, which evidences some internal differences between C0/C4 and C8. The reason why we do not observe a correlation between hydrophobicity and length could be that C8 has a different fiber internal arrangement.^33^

A temperature-ramped CD, measured on the inner azobenzene range, provides interesting insights into the fiber stacking and its temperature dependency (Figure 2b). C0 and C4 share the same temperature response; a positive Cotton effect can be observed at low temperatures that inverts reversibly to a negative Cotton effect at high temperatures. This behavior was previously associated with an azobenzene stacking rearrangement and an increased stability state. It is originated from the loss of solvation water of the OEG at high temperatures, increasing hydrophobicity and reducing steric hindrance.^34,35^ On the other hand, C8 shows a permanent negative Cotton effect at all temperatures, suggesting a different azobenzene arrangement and a superior stability. C8 only shows a CD signal decrease, probably deriving from partial depolymerization at higher temperatures.^36^ C0 and C4 start showing a decrease in CD intensity above 50 °C; however, none of them disassemble completely.

FRET experiments, performed at 37 °C, with 10% mol of labeled monomer (5% sCy3 and 5% sCy5) also show differences between the molecules under the same conditions (Figure 2c). C0 shows a lower FRET signal (∼0.5), C4 a moderate signal (∼2.5), and C8 a surprisingly high signal (∼23). This trend matches the hydrophobic–hydrophilic balance trend of the molecules, and it is explained by a combination of two parameters: the distance and the number of the FRET pair of dyes. As C0 is the least hydrophobic of the three, the free monomer concentration is probably higher, meaning that less monomer is assembled and, hence, less FRET is obtained. However, the C8 signal seems extraordinarily high to be generated only by this effect. We hypothesize that the different internal arrangement of C8 fibers put dyes in closer proximity leading to the increased energy transfer, since it scales with *R*^–6^.

Overall, the three monomers self-assemble into fibers. C0 and C4 fibers are morphologically similar, and the azobenzene moieties reorganize at high temperatures. C8 fibers are thicker and are not responsive to temperature. Finally, C0, C4, and C8 exhibit a FRET ratio with quantitative differences that match their respective degree of aggregation.

### 2.3 Light-Responsive Abilities

**Figure 3:**
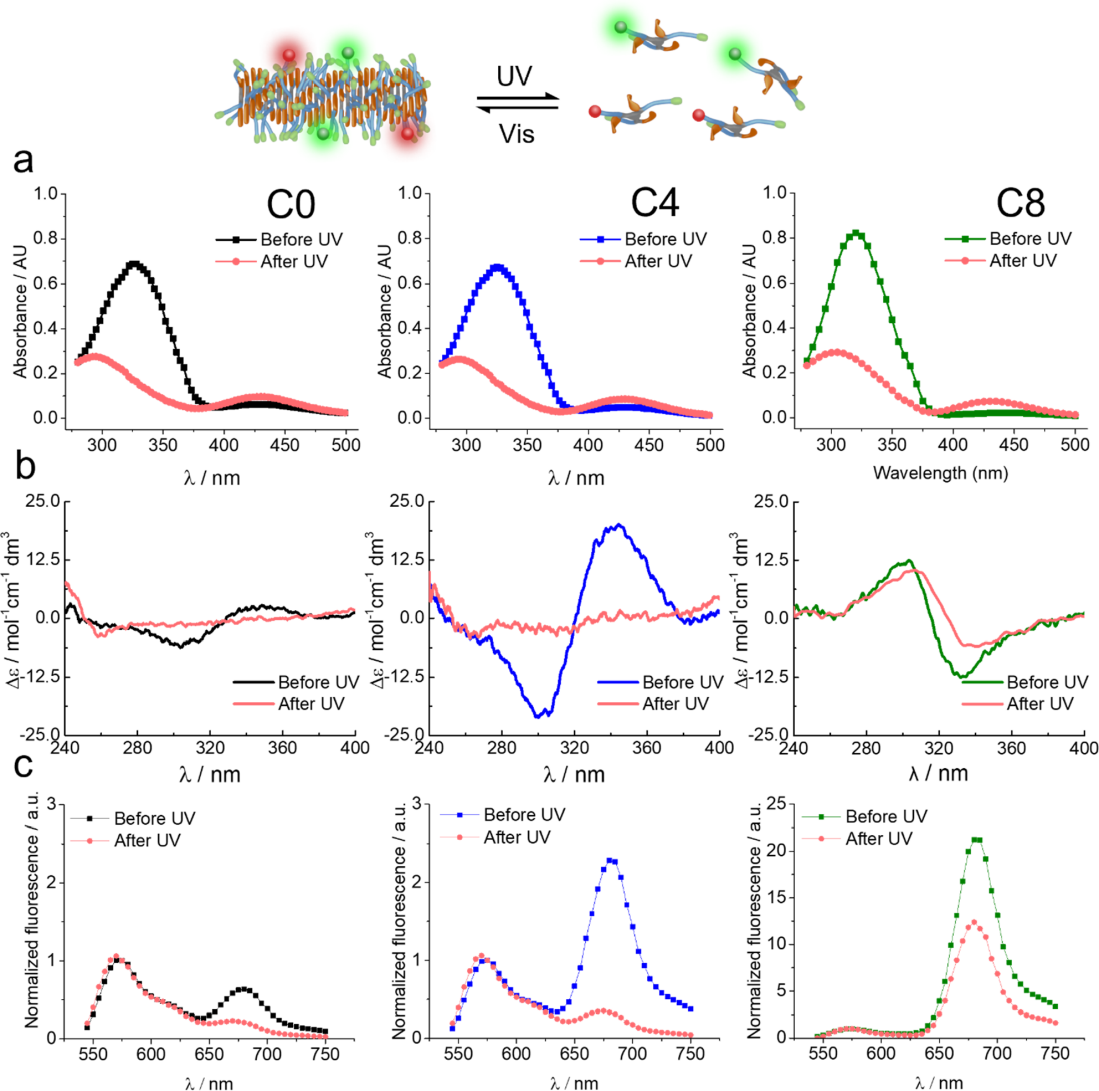
(a) *E*–*Z* photoisomerization and (b) CD spectra of monomers at 25 μM before and after irradiation in water. (c) FRET spectra in PBS and at 37 °C before and after irradiation, matching the CD results (C0, left column; C4, middle column; C8, right column) (line was added to guide the eye). Irradiation conditions: 365 nm, 1000 mA at 100% of the LED intensity, where irradiation time was modified in each case to reach plateau: UV–vis/CD (cuvette): 8 s for C0, 10 s for C4, and 93 s for C8. FRET (microplate): 30 s for C0 and C4, 360 s for C8. Kinetics of isomerization was obtained for each molecule (Figures S15 and S16).

For the sake of establishing the stability–responsiveness balance of our systems in vitro, the light-responsive abilities must be tested. It is of interest to understand if fibers of increasing hydrophobic blocks can be disassembled by azobenzene photoisomerization. We studied the samples before and after UV irradiation by UV–vis and CD spectroscopy, FRET, and TEM (Figure 3 and SI).

The absorption spectra show that the azobenzene moieties in all three cases are initially in *E* configuration (330 nm peak) and then isomerize to *Z* configuration (450 nm peak) after irradiation with UV light (360 nm). It is interesting to mention that C8 presents few differences. The absorption band attributed to the *E* isomer (∼330 nm) is blue shifted by ∼5 nm, the population of initial *Z* isomers is slightly lower, and the kinetics of photoisomerization is slower. In Figures S15 and S16, we can compare the photo-isomerization kinetics of the three molecules in the assembled (H_2_O/PBS) and molecularly dissolved states (DMSO). C0 and C4 displayed the same behavior in water than in DMSO. However, C8 showed a decreased extent of photoisomerization only in water (monomers assembled), while in DMSO, the response was identical to C0 and C4. In other words, C8 needed to be irradiated longer to achieve the same extent of isomerization. Most likely, isomerization of C8 in the assembled state is less favored because of the tight packing and steric effects in such strongly bound assemblies.^37,38^ In Figure S18, the reversibility and photofatigue resistance of the system is demonstrated.

CD and FRET give insights about the self-assembly state of the monomers (Figure 3). Before irradiation, each monomer shows its own characteristic CD signal. After irradiation, the CD signal reaches noise levels for C0 and C4, suggesting depolymerization of the supramolecular fibers.^27^ For C8, the signal is reduced but it is still present. FRET experiments match CD results and follow the same trend: C0 and C4 signals decrease to minimum levels, while C8 decreases partially. This shows that FRET is an excellent technique to monitor self-assembly. These results have been further confirmed by TEM (Figure S19), where irradiated C0 and C4 did not show fibers, while irradiated C8 showed a shortening of the fibers, reaching lengths of ∼35 nm.

We can conclude that C0 and C4 are fully responsive to irradiation with UV light, disassembling completely, while C8 is partially responsive. C8 needed higher irradiation times to isomerize, and still short assemblies remain. The azobenzene isomerization represents a massive change for a stacked system in terms of space, orientation, and symmetry. For this reason, it is an adequate approach to trigger disassembly even in very hydrophobic systems that can show insensitivity to other external cues. Hence, it is a convenient strategy to define the stability/responsiveness trade-off of the system, before evaluating the robustness in the biological environment.

### 2.4 Supramolecular Dynamics

**Figure 4:**
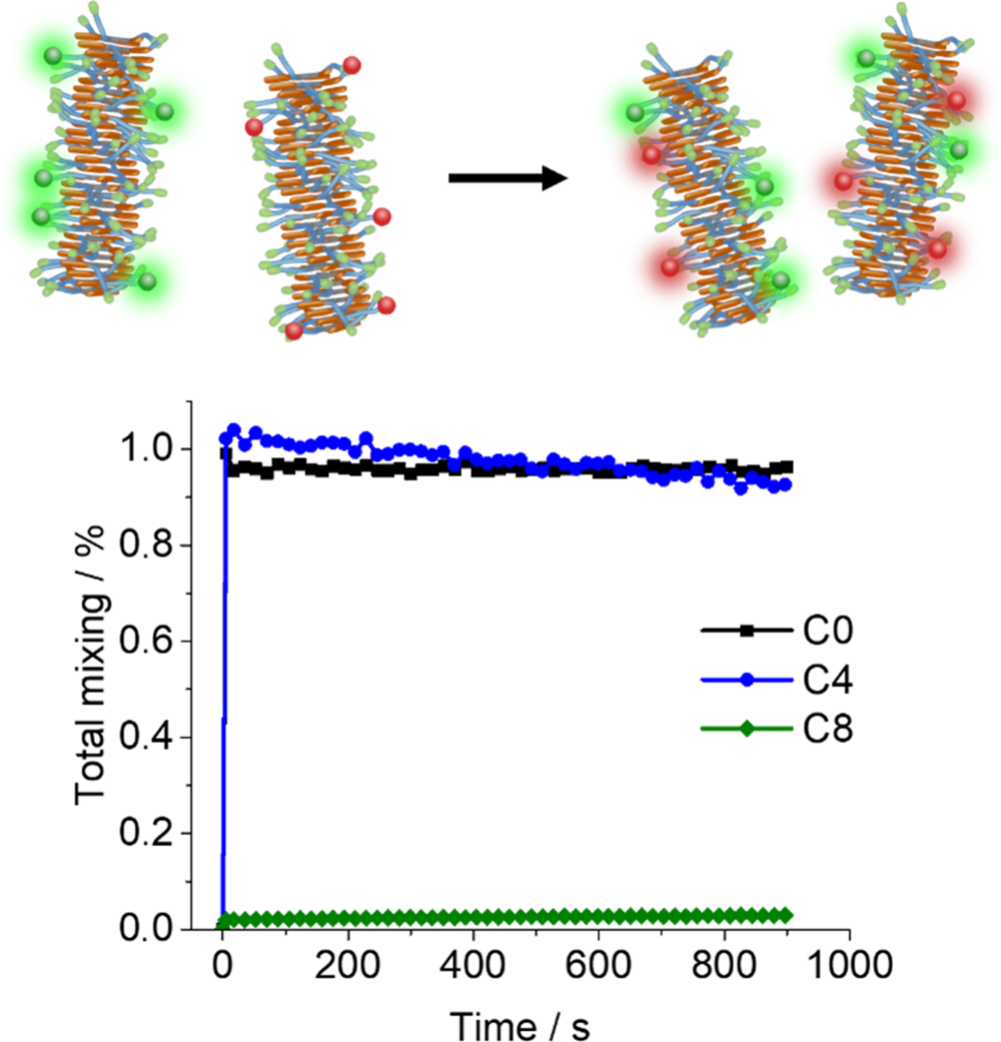
Mixing of 25 μM 10% sCy3-labeled fibers with 25 μM 10% sCy5 fibers at 37 °C, monitored in time. After mixing, the labeled monomer content is 10%, 5% for sCy3, and 5% for sCy5, as shown in Figures 2 and 3. A line was added to guide the eye. Total mixing was defined relatively to analogous FRET experiments, where the components were formulated together from the beginning and not mixed after formulation.

The dynamics of supramolecular polymers can be assessed by the velocity of exchange between assembled and free monomers. Mixing fibers labeled with different fluorophores (out of equilibrium) and letting it equilibrate to homogeneously labeled fibers (equilibrium) give an idea of how fast assembled monomers exchange with the solvent, or if they exchange at all. Two solutions of fibers were prepared, the first containing 10% mol of sCy3-labeled monomer and the second containing 10% mol of sCy5-labeled monomer. After mixing the solutions together, a final 10% mol labeling was achieved (5% of each probe), and the FRET ratio was monitored over time (Figure 4).

Once again, for C0 and C4, the results are similar. FRET shows maximum values immediately after mixing. After that, they present a slight decrease with time, but it does not seem significant. On the other side, C8 presents a negligible increase. These results suggest that C8 is governed by kinetics, displaying a very slow monomer exchange (low *k*_off_).^16,39^

This huge difference between C0/C4 and C8 behaviors anticipates a high difference in stability, given that C0/C4 are highly dynamic (monomers exchange quickly), while C8 is highly static (negligible monomer exchange). For this reason, C0 and C4 will be more sensitive to changes in the free monomer concentration than C8.

### 2.5 Behavior in Biological Media

Supramolecular drug delivery carriers must resist harsh conditions upon intravenous injection.^40^ Five thousand-fold dilutions are faced on top of potential side interactions with serum proteins in large excess. Thus, understanding the self-assembly dependencies on dilution and protein concentration is of major importance.

Using FRET, we assessed the assembly degree of the systems and compared the behavior of a one-half serial dilution in PBS and with an increasing concentration of bovine serum albumin (BSA) assay. We aim to understand how these two factors promote disassembly of fibers of different stability.

**Figure 5:**
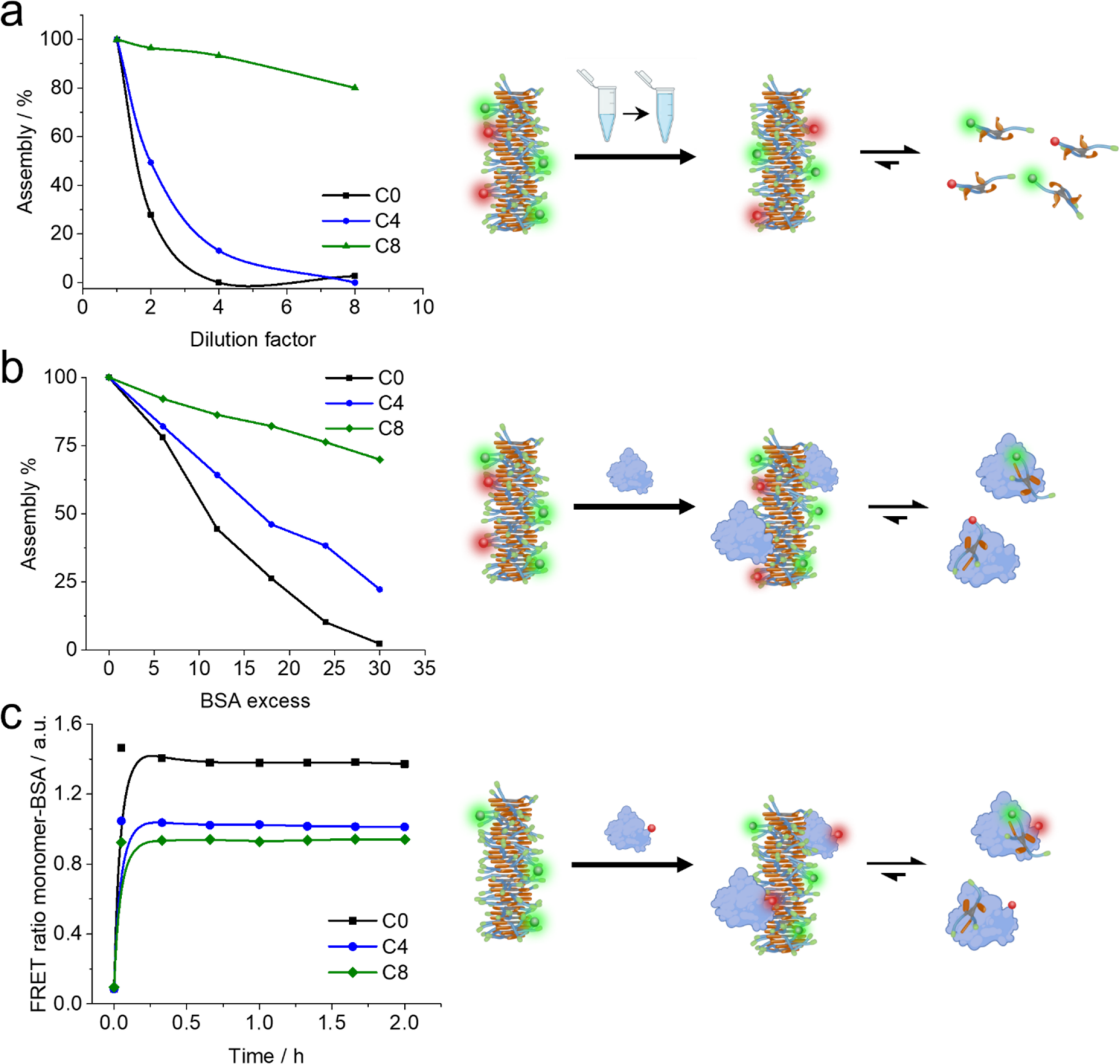
(a) Supramolecular stability along a one-half serial dilution experiment from 25 μM samples at 37 °C. (b) Supramolecular stability vs [BSA] of samples at 25 μM. (c) FRET ratio of the polymer–BSA interaction vs time, at a concentration of 25 μM, 10 mg/mL of BSA, and 10% labeled (a line was added to guide the eye).

Dilution experiments showed two different results, matching the stability/responsiveness balance (Figure 5a). On one side, C0 and C4 showed a dramatic decrease in the population of self-assembled structures upon dilution. On the other side, C8 showed a very mild decrease. The results show that C0 and C4 cannot remain assembled upon dilution, while C8 is mildly affected in this range. Interestingly, FRET decreased rapidly in time in all the cases, even for C8. This effect on C8 seems opposite to the dynamic’s experiments in vitro (Figure 3), in which we could see a very slow monomer exchange. As release of the monomer from the C8 fiber is not a likely explanation, other concentration-dependent phenomena like fiber bundling could be the origin of this mild effect. Altogether, we observe two well-differentiated regimes, low stability and high responsiveness and high stability and low responsiveness.

Then, polymers were incubated with an increasing concentration of BSA, mimicking a hypothetical intravenous injection. Results showed the same C0–C8 trend as before, but with important differences (Figure 5b). Here, the protein clearly destabilized all the monomers, with C0 being the most affected due to the low robustness and C8 being the less affected thanks to its outstanding robustness. This destabilization occurs via monomer sequestration by BSA.^18^ We demonstrated it experimentally by labeling BSA with Cy5 dye and mixing it with supramolecular polymers loaded only with sCy3. FRET was obtained for the three molecules, proving the interaction (Figure 5c). This time, the FRET ratio is lower for C8 (less disassembly caused by BSA) and higher for C0 (higher disassembly caused by BSA). In addition, we irradiated the samples with UV to disassemble the polymers and observe whether the BSA is interacting with monomers or fibers. We could observe that the FRET signal after UV irradiation (disassembly) did not change significantly, indicating that the signal is primarily coming from BSA bound to monomers (Figure S23). Although demonstrating the scavenging effect of BSA over monomers, it remains unknown which route of disassembly it takes.

**Figure 6:**
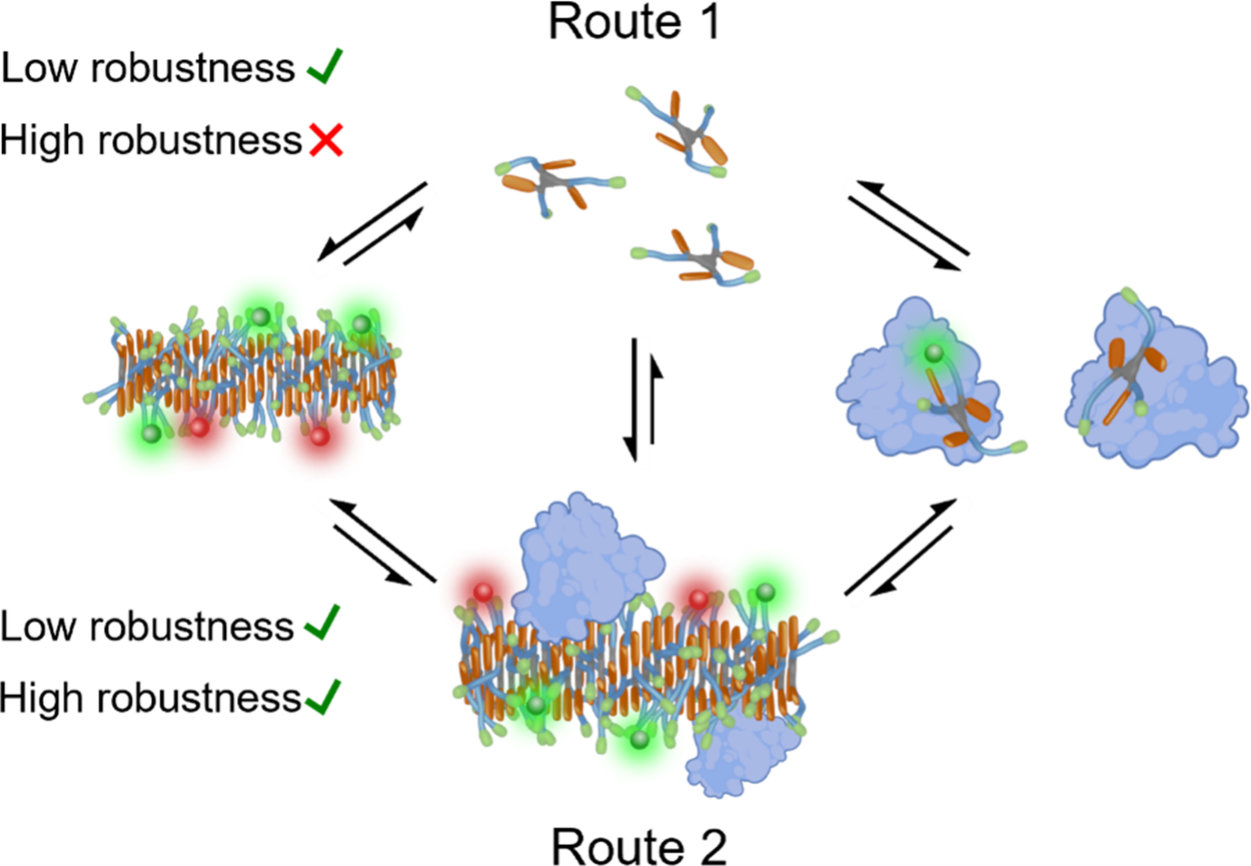
Cartoon representing the possible disassembling pathways. Route 1, protein scavenging from free monomer, at the top. Route 2, protein scavenging from fiber, at the bottom. Route 1 is forbidden for highly stable assemblies.

As demonstrated by a recent work of the group of Meijer, BSA can interact with both free and assembled BTA monomers.^20^ They demonstrated using computational simulations that monomers can diffuse toward the protein when fiber and protein are in direct contact, and it requires less energy than doing it as free monomers. For this reason, our supramolecular polymers can be destabilized following two interaction pathways (Figure 6):

1. BSA scavenges the free monomers in solution, depleting the free monomer concentration and forcing the release of monomers from the fibers to maintain the concentration. The destabilization is governed by the *k*_off_ of the monomers.
2. BSA scavenges monomers from the fibers, after direct contact. The disassembly is governed by the *K*_d_ of the BSA fiber.

The first situation just requires a very robust structure displaying a kinetic stability, to remain stable under out-of-equilibrium conditions (depleted free monomer). The stability in the second situation is unrelated to the monomer–fiber equilibrium and is directly dependent on the BSA–fiber interaction.

Our results give clear insights regarding this point. The disassembly of C8 for both dilution and BSA experiments is similar in absolute terms but not in relative terms compared to C0/C4. The effect of proteins on C8 relative to C0 and C4 is much higher than the dilution effect. In other words, the difference between C0/C4 and C8 is dramatic for dilution and quite mild for proteins. Where before we had a clear difference between the two stability/responsiveness regimes, now the difference is very diffuse. This necessarily implies that C8 is more sensitive to the presence of proteins than to dilution. Very importantly, C8 does not exchange monomers at a significant rate as observed in the mixing experiment (Figure 4). Then, for C8, BSA must scavenge monomers directly from the fibers, route 2 (Figure 6). For C0 and C4, the same interaction mechanism should exist, however, we cannot discard the disassembly through the free monomer scavenging (route 2).

### 2.6 Discussion

We demonstrated experimentally that the assembled monomer state and the monomer–BSA state are connected through two different routes, rather than being connected only through the free monomer. Even though it is not clear whether low-stability polymers can lose monomers using both routes or not, high-stability polymers can exclusively be destabilized by the direct interaction with proteins. This implies that stability in the biological environment is achieved not only by high supramolecular robustness but also by absence of scavenging effects. Hence, the protein–supramolecule interaction is decisive for the stability. When it is high, proteins would interact longer, eventually allowing the monomer diffusion toward the BSA.^20^ Once bound, BSA could detach from the fiber depleting the monomer from the fiber, leaving the stability–responsiveness balance in a second plane. If, just for the sake of speculation, we assume a linear trend for the C8 assembly vs BSA excess graph (Figure 5b), we can extrapolate that complete disassembly would happen at 95-fold excess of protein. Upon a hypothetical intravenous injection, this would represent only around 0.7% of the actual albumin in the blood. Proteins are in vast number; if they can bind fibers, they will eventually deplete all the monomers. For this reason, and because proteins in the blood outnumber monomers by several orders of magnitude, the key factor for in-blood stability falls on reducing the protein interaction rather than increasing the stability–responsiveness balance.

In scenarios where the protein–supramolecule interaction is low, proteins interact only with free monomers, and the only requirement for in-blood stability is high supramolecular stabilities, typically associated with kinetic effects and low CAC values.^41,42^ This reveals the importance of studying the protein–supramolecule interactions.

Overall, works on micelles have shown a result in accordance with our observation. Robust micelles with low CMC from the Thayumanavan group containing inner enzyme cleavable groups have shown cleavage ONLY when the protein specifically binds to a ligand on the surface.^12^ Analogous micelles from the Amir group containing inner enzyme cleavable groups have shown no cleavage in the presence of proteins.^18^ Both examples show how high stability hampers the release of monomers to re-equilibrate for replacing the cleaved free monomer (route 1). However, in the presence of proteins that specifically bind to the surface, the cleavage is possible. Proteins can bind and scavenge monomers that get exposed to the enzyme (route 2). When this interaction appears to be weak, the presence of proteins has a minimal effect.

In future works, the stability of the supramolecular polymers in the presence of cells and in complex media will have to be addressed. To do so, microscopy techniques could be useful to assess the spectroscopic signature of individual polymers. For example, FRET could be observed in confocal mode by detecting donor and acceptor signal in two separate channels.^19^ Additionally, assays could potentially benefit from super-resolution modes like for example STED microscopy (similarly to confocal) or combining FRET with DNA-PAINT as previously reported by Auer et al.^43^ While these techniques are not easy to implement, the potential information we could obtain regarding the stability and performance of supramolecular polymers in cells is vast.

## 3 Conclusions

We have synthesized three BTA-based discotic amphiphiles, with three different hydrophobic–hydrophilic balances. The evaluation of their stability–responsiveness trade-off allowed to separate them into two well-differentiated regimes, low stability–high responsivity for C0 and C4 and high stability–low responsivity for C8.

In a hypothetical intravenous injection, C0 and C4 clearly showed very low stability. Dilution seems critical for these structures, and protein scavenging monomers also leads to disassembly.

C8 forms an extraordinarily robust and static supramolecular polymer, whose responsiveness is already compromised, which is stable against dilution. However, proteins seem to destabilize the structures. This is possible only because proteins can scavenge monomers directly from fibers and not only from solution. For this reason, even this highly robust and static polymer would not be suitable for systemic drug delivery applications. We demonstrate that designing supramolecules for drug delivery requires inevitably a high stability–responsiveness trade-off to resist dilution and a weak BSA–supramolecule interaction to minimize scavenging effects.

## Supporting Information Available

Experimental procedure for the synthesis of C0, C4, and C8 as well as analytical, spectral, and self-assembly characterization data (PDF)

## Abbreviations

TEM: transmission electron microscopy
CD: circular dichroism
HPLC: high-performance liquid chromatography
BTA: benzene-1,3,5-tricarboxamide
SPPS: solid phase peptide synthesis
Fmoc: fluorenylmethyloxycarbonyl
Boc: *tert*-butyloxycarbonyl
DMSO: dimethyl sulfoxide
BSA: bovine serum albumin
FRET: Förster resonance energy transfer

## References

[ref1] Nogales E. Structural Insights into Microtubule Function. Annu. Rev. Biochem. 2000, 69, 277–302. 10.1146/annurev.biochem.69.1.277. 10966460

[ref2] Vale R. D. The Molecular Motor Toolbox for Intracellular Transport. Cell 2003, 112, 467–480. 10.1016/S0092-8674(03)00111-9. 12600311

[ref3] Forth S.; Kapoor T. M. The Mechanics of Microtubule Networks in Cell Division. J. Cell Biol. 2017, 216, 1525–1531. 10.1083/jcb.201612064. 28490474 PMC5461028

[ref4] Pollard T. D.; Cooper J. A. Actin, a Central Player in Cell Shape and Movement. Science 2009, 326, 1208–1212. 10.1126/science.1175862. 19965462 PMC3677050

[ref5] Krieg E.; Bastings M. M. C.; Besenius P.; Rybtchinski B. Supramolecular Polymers in Aqueous Media. Chem. Rev. 2016, 116, 2414–2477. 10.1021/acs.chemrev.5b00369. 26727633

[ref6] Studart A. R. Biologically Inspired Dynamic Material Systems. Angew. Chem., Int. Ed. 2015, 54, 3400–3416. 10.1002/anie.201410139. 25583299

[ref7] Ma X.; Tian H. Stimuli-Responsive Supramolecular Polymers in Aqueous Solution. Acc. Chem. Res. 2014, 47, 1971–1981. 10.1021/ar500033n. 24669851

[ref8] Freeman R.; Han M.; Álvarez Z.; Lewis J. A.; Wester J. R.; Stephanopoulos N.; McClendon M. T.; Lynsky C.; Godbe J. M.; Sangji H.; Luijten E.; Stupp S. I. Reversible Self-Assembly of Superstructured Networks. Science 2018, 362, 808–813. 10.1126/science.aat6141. 30287619 PMC6420308

[ref9] Albertazzi L.; Martinez-Veracoechea F. J.; Leenders C. M. A.; Voets I. K.; Frenkel D.; Meijer E. W. Spatiotemporal Control and Superselectivity in Supramolecular Polymers Using Multivalency. PNAS 2013, 110, 12203–12208. 10.1073/pnas.1303109110. 23836666 PMC3725081

[ref10] Proetto M. T.; Callmann C. E.; Cliff J.; Szymanski C. J.; Hu D.; Howell S. B.; Evans J. E.; Orr G.; Gianneschi N. C. Tumor Retention of Enzyme-Responsive Pt(II) Drug-Loaded Nanoparticles Imaged by Nanoscale Secondary Ion Mass Spectrometry and Fluorescence Microscopy. ACS Cent. Sci. 2018, 4, 1477–1484. 10.1021/acscentsci.8b00444. 30555899 PMC6276039

[ref11] Harnoy A. J.; Slor G.; Tirosh E.; Amir R. J. The Effect of Photoisomerization on the Enzymatic Hydrolysis of Polymeric Micelles Bearing Photo-Responsive Azobenzene Groups at Their Cores. Org. Biomol. Chem. 2016, 14, 5813–5819. 10.1039/C6OB00396F. 27093537

[ref12] Guo J.; Zhuang J.; Wang F.; Raghupathi K. R.; Thayumanavan S. Protein AND Enzyme Gated Supramolecular Disassembly. J. Am. Chem. Soc. 2014, 136, 2220–2223. 10.1021/ja4108676. 24447098 PMC3985736

[ref13] Liu B.; Thayumanavan S. Substituent Effects on the PH Sensitivity of Acetals and Ketals and Their Correlation with Encapsulation Stability in Polymeric Nanogels. J. Am. Chem. Soc. 2017, 139, 2306–2317. 10.1021/jacs.6b11181. 28106385 PMC5382500

[ref14] Choi W.; Battistella C.; Gianneschi N. C. High Efficiency Loading of Micellar Nanoparticles with a Light Switch for Enzyme-Induced Rapid Release of Cargo. Biomater. Sci. 2021, 9, 653–657. 10.1039/D0BM01713B. 33300507 PMC9753762

[ref15] Spitzer D.; Rodrigues L. L.; Straßburger D.; Mezger M.; Besenius P. Tuneable Transient Thermogels Mediated by a PH- and Redox-Regulated Supramolecular Polymerization. Angew. Chem., Int. Ed. 2017, 56, 15461–15465. 10.1002/anie.201708857. 29044991

[ref16] Krieg E.; Rybtchinski B. Noncovalent Water-Based Materials: Robust yet Adaptive. Chem. – Eur. J. 2011, 17, 9016–9026. 10.1002/chem.201100809. 21726009

[ref17] Othman Z.; Cillero Pastor B.; van Rijt S.; Habibovic P. Understanding Interactions between Biomaterials and Biological Systems Using Proteomics. Biomaterials 2018, 167, 191–204. 10.1016/j.biomaterials.2018.03.020. 29571054

[ref18] Feiner-Gracia N.; Buzhor M.; Fuentes E.; Pujals S.; Amir R. J.; Albertazzi L. Micellar Stability in Biological Media Dictates Internalization in Living Cells. J. Am. Chem. Soc. 2017, 139, 16677–16687. 10.1021/jacs.7b08351. 29076736

[ref19] Feiner-Gracia N.; Glinkowska Mares A.; Buzhor M.; Rodriguez-Trujillo R.; Samitier Marti J.; Amir R. J.; Pujals S.; Albertazzi L. Real-Time Ratiometric Imaging of Micelles Assembly State in a Microfluidic Cancer-on-a-Chip. ACS Appl. Bio Mater. 2021, 4, 669–681. 10.1021/acsabm.0c01209. PMC7818510 33490884

[ref20] Varela-Aramburu S.; Morgese G.; Su L.; Schoenmakers S. M. C.; Perrone M.; Leanza L.; Perego C.; Pavan G. M.; Palmans A. R. A.; Meijer E. W. Exploring the Potential of Benzene-1,3,5-Tricarboxamide Supramolecular Polymers as Biomaterials. Biomacromolecules 2020, 21, 4105–4115. 10.1021/acs.biomac.0c00904. 32991162 PMC7556542

[ref21] Zana R. Critical Micellization Concentration of Surfactants in Aqueous Solution and Free Energy of Micellization. Langmuir 1996, 12, 1208–1211. 10.1021/la950691q.

[ref22] Van Domeselaar G. H.; Kwon G. S.; Andrew L. C.; Wishart D. S. Application of Solid Phase Peptide Synthesis to Engineering PEO–Peptide Block Copolymers for Drug Delivery. Colloids Surf., B 2003, 30, 323–334. 10.1016/S0927-7765(03)00125-5.

[ref23] Owen S. C.; Chan D. P. Y.; Shoichet M. S. Polymeric Micelle Stability. Nano Today 2012, 7, 53–65. 10.1016/j.nantod.2012.01.002.

[ref24] Rajdev P.; Ghosh S. Fluorescence Resonance Energy Transfer (FRET): A Powerful Tool for Probing Amphiphilic Polymer Aggregates and Supramolecular Polymers. J. Phys. Chem. B 2019, 123, 327–342. 10.1021/acs.jpcb.8b09441. 30407823

[ref25] Besenius P.; Portale G.; Bomans P. H. H.; Janssen H. M.; Palmans A. R. A.; Meijer E. W. Controlling the Growth and Shape of Chiral Supramolecular Polymers in Water. Proc. Natl. Acad. Sci. 2010, 107, 17888–17893. 10.1073/pnas.1009592107. 20921365 PMC2964246

[ref26] Leenders C. M. A.; Albertazzi L.; Mes T.; Koenigs M. M. E.; Palmans A. R. A.; Meijer E. W. Supramolecular Polymerization in Water Harnessing Both Hydrophobic Effects and Hydrogen Bond Formation. Chem. Commun. 2013, 49, 1963–1965. 10.1039/C3CC38949A. 23364450

[ref27] Fuentes E.; Gerth M.; Berrocal J. A.; Matera C.; Gorostiza P.; Voets I. K.; Pujals S.; Albertazzi L. An Azobenzene-Based Single-Component Supramolecular Polymer Responsive to Multiple Stimuli in Water. J. Am. Chem. Soc. 2020, 142, 10069–10078. 10.1021/jacs.0c02067. 32395995 PMC7497294

[ref28] Aemissegger A.; Hilvert D. Synthesis and Application of an Azobenzene Amino Acid as a Light-Switchable Turn Element in Polypeptides. Nat. Protoc. 2007, 2, 161–167. 10.1038/nprot.2006.488. 17401350

[ref29] Leenders C. M. A.; Baker M. B.; Pijpers I. A. B.; Lafleur R. P. M.; Albertazzi L.; Palmans A. R. A.; Meijer E. W. Supramolecular Polymerisation in Water; Elucidating the Role of Hydrophobic and Hydrogen-Bond Interactions. Soft Matter 2016, 12, 2887–2893. 10.1039/C5SM02843D. 26892482 PMC4849209

[ref30] Roy R.; Hohng S.; Ha T. A Practical Guide to Single Molecule FRET. Nat. Methods 2008, 5, 507–516. 10.1038/nmeth.1208. 18511918 PMC3769523

[ref31] Algar W. R.; Hildebrandt N.; Vogel S. S.; Medintz I. L. FRET as a Biomolecular Research Tool — Understanding Its Potential While Avoiding Pitfalls. Nat. Methods 2019, 16, 815–829. 10.1038/s41592-019-0530-8. 31471616

[ref32] Baker M. B.; Albertazzi L.; Voets I. K.; Leenders C. M. A.; Palmans A. R. A.; Pavan G. M.; Meijer E. W. Consequences of Chirality on the Dynamics of a Water-Soluble Supramolecular Polymer. Nat. Commun. 2015, 6, 6234 10.1038/ncomms7234. 25698667 PMC4346625

[ref33] Lafleur R. P. M.; Herziger S.; Schoenmakers S. M. C.; Keizer A. D. A.; Jahzerah J.; Thota B. N. S.; Su L.; Bomans P. H. H.; Sommerdijk N. A. J. M.; Palmans A. R. A.; Haag R.; Friedrich H.; Böttcher C.; Meijer E. W. Supramolecular Double Helices from Small C3-Symmetrical Molecules Aggregated in Water. J. Am. Chem. Soc. 2020, 142, 17644–17652. 10.1021/jacs.0c08179. 32935541 PMC7564094

[ref34] Smith G. D.; Bedrov D. Roles of Enthalpy, Entropy, and Hydrogen Bonding in the Lower Critical Solution Temperature Behavior of Poly(Ethylene Oxide)/Water Solutions. J. Phys. Chem. B 2003, 107, 3095–3097. 10.1021/jp0270046.

[ref35] Lüsse S.; Arnold K. The Interaction of Poly(Ethylene Glycol) with Water Studied by 1H and 2H NMR Relaxation Time Measurements. Macromolecules 1996, 29, 4251–4257. 10.1021/ma9508616.

[ref36] Hartlieb M.; Mansfield E. D. H.; Perrier S. A Guide to Supramolecular Polymerizations. Polym. Chem. 2020, 11, 1083–1110. 10.1039/C9PY01342C.

[ref37] Bandara H. M. D.; Burdette S. C. Photoisomerization in Different Classes of Azobenzene. Chem. Soc. Rev. 2012, 41, 1809–1825. 10.1039/C1CS15179G. 22008710

[ref38] Valley D. T.; Onstott M.; Malyk S.; Benderskii A. V. Steric Hindrance of Photoswitching in Self-Assembled Monolayers of Azobenzene and Alkane Thiols. Langmuir 2013, 29, 11623–11631. 10.1021/la402144g. 23924041

[ref39] Cui H.; Chen Z.; Zhong S.; Wooley K. L.; Pochan D. J. Block Copolymer Assembly via Kinetic Control. Science 2007, 317, 647–650. 10.1126/science.1141768. 17673657

[ref40] Chenthamara D.; Subramaniam S.; Ramakrishnan S. G.; Krishnaswamy S.; Essa M. M.; Lin F.-H.; Qoronfleh M. W. Therapeutic Efficacy of Nanoparticles and Routes of Administration. Biomater. Res. 2019, 23, 20 10.1186/s40824-019-0166-x. 31832232 PMC6869321

[ref41] Su H.; Wang F.; Ran W.; Zhang W.; Dai W.; Wang H.; Anderson C. F.; Wang Z.; Zheng C.; Zhang P.; Li Y.; Cui H. The Role of Critical Micellization Concentration in Efficacy and Toxicity of Supramolecular Polymers. Proc. Natl. Acad. Sci. 2020, 117, 4518–4526. 10.1073/pnas.1913655117. 32071209 PMC7060728

[ref42] Ma S.; Zhou J.; Zhang Y.; He Y.; Jiang Q.; Yue D.; Xu X.; Gu Z. Highly Stable Fluorinated Nanocarriers with iRGD for Overcoming the Stability Dilemma and Enhancing Tumor Penetration in an Orthotopic Breast Cancer. ACS Appl. Mater. Interfaces 2016, 8, 28468–28479. 10.1021/acsami.6b09633. 27712073

[ref43] Auer A.; Strauss M. T.; Schlichthaerle T.; Jungmann R. Fast, Background-Free DNA-PAINT Imaging Using FRET-Based Probes. Nano Lett. 2017, 17, 6428–6434. 10.1021/acs.nanolett.7b03425. 28871786

