# Supplementary material for: Supramolecular Stability of Benzene-1,3,5-tricarboxamide Supramolecular Polymers in Biological Media: Beyond the Stability–Responsiveness Trade-off
Source: J Am Chem Soc. 2022 Nov 11;144(46):21196–205. doi: 10.1021/jacs.2c08528 (PMC9706554; doi:10.1021/jacs.2c08528)
Supplement: Supplementary file 1 — ja2c08528_si_001.pdf [file ja2c08528_si_001.pdf]

Supporting Information (SI)

Supramolecular stability of benzene-1,3,5-tricarboxamide supramolecular polymers in biological media: beyond the stability-responsiveness trade-off

Edgar Fuentes, Yeray Gabaldón, Mario Collado, Shikha Dhiman, José Augusto Berrocal, Sílvia Pujals*and Lorenzo Albertazzi*

Contents

1. MATERIALS **2**

2. INSTRUMENTATION **2**

3. CHEMICAL SYNTHESIS **3**

4. SELF-ASSEMBLY AND UV RESPONSE **8**

5. STABILITY IN BIOLOGICAL-LIKE CONDITIONS EXPERIMENTS **9**

1. Materials

All solvents, unless stated otherwise, were obtained from commercial sources in at least analytical quality (a.r.) and were used without further purification. Ultrapure water was obtained from a Milli Pore system from Merck. Lysine and 2-Chlorotrityl chloride resin were obtained from Iris biotech. Fmoc-PEG_8-OH and PyBOP were purchased from Chempep. DIEA, piperidine, formic acid benzene tricarbonyl chloride, Fmoc-GABA and Fmoc-8-Aoc-OHwere obtained from Sigma-Aldrich.

L-Phenylalanine-4'-azobenzene was synthesised following the procedure reported before[1], [2].

2. Instrumentation

UV-Vis: Measurements were performed with a Tecan infinite M200Pro microplate reader using a quartz cuvette of 0.5 mL and 1 cm of pathlength. Spectra were taken from 280 nm to 500 nm at every 5 nm.

Fluorescence: Measurements were performed with a Tecan infinite M200Pro microplate reader using a 96 well-plate, and 150 µL of sample. Spectra were taken from 550 nm to 750 nm at every 5 nm, exciting at 510 nm and using an integration time of 20 µs, 18 flashes and gain of 120.

Reverse Phase-High Performance Liquid Chromatography: During synthesis and characterization of the monomers, measurements were recorded on a HPLC Waters e2695 separation module equipped with Photodiode Array detector Waters 2998 and Mass spectrometer QDA detector Acquity. A XSelect CSH C18 3.5 µm 4.6x50mm was used. Water 0.1% HCOOH and ACN 0.1% HCOOH were used as eluents. A gradient of 25-45% ACN in water was applied in 3.5 min. The elution speed was kept at 1.6 ml·min^{-1} and the temperature was maintained at 50 °C. The injection volume was 10 µl.

For labelled monomers, a gradient of 20-60% ACN in water was applied in 3.5 min. The elution speed was kept at 1.6 ml·min^{-1} and the temperature was maintained at 50 °C. The injection volume was 5 µl.

Transmission Electron Microscopy: Images were taken with Jeol JEM 1010 MT electron microscope (Japan), operating at 80 kV. Images were obtained on a CCD camera Megaview III (ISIS), MNnster, Germany.

Negative staining: Samples were deposited onto C-only grids (pre-treated with glow discharge for 30 s) for 1.5 min. The solution was washed with water for 30 s, and finally it was negatively stained with uranyl acetate 2% during 1.5 min. The excess of solution was blotted with a filtering paper and the grids were stored in the desiccator.

Circular Dichroism: spectra was recorded on a Jasco J815 spectrometer, equipped with a JASCO Peltier PFD-425S/15 with a range of 263–383 K. A sealable quartz cell with a pathlength of 5 mm was used. The spectra were recorded continuously between 500-240 nm at every 0.1 nm, with a sensitivity of 100 mdeg, at 100 nm/min. The response time was set to 0.25s. The temperature dependent spectra were recorded at an interval of 10 °C between 5-75 °C at a temperature ramp of 1 °C/min. During photoirradiation measurements the sample was kept isothermal at 20 °C.

^{1}H-NMR: ^{1}H NMR spectra were recorded on a Varian Mercury Vx 400 MHz. Chemical shifts are given in ppm (δ) values relative to tetramethylsilane (TMS).

Maldi-TOF: spectra were recorded on a Applied Biosystems - 4800 Plus MALDI TOF/TOF™ Analyzer. α-Cyano-4-hydroxycinnamic acid was used as a matrix.

3. Experimental section

3.1 Chemical synthesis

The synthetic strategy consists of growing the wedge on a polymeric support (Solid Phase Peptide Synthesis), the subsequent cleavage from the solid and the final convergent coupling to the core in solution. The synthesis of C0 was previously reported[3].

- **Solid-phase synthesis of C4 and C8 wedges**

Resin swelling: 2-chloro-trytil chloride resin (1.6 mmol/g) was transferred to a reaction syringe and swelled first with DCM and next with DMF, following:

I. DCM: x5 washings of 15 mL + 15 mL for 1 h.
II. DMF: x5 washings of 15 mL + 15 mL for 30 min.
III. DCM: x5 washings of 15 mL + 15 mL for 1 h

Loading: Just after swelling, the first aa (Lys) was coupled. 1 eq Fmoc-Lys(Boc)-OH, 3 eq DIEA (N,N-Diisopropylethylamine) in 5 mL DCM for 1 h (manual shaking the first 5 minutes). MeOH (1 mL) was added to the mixture for 10 min. Then, the contents of the syringe were filtered and washed with DCM 15 mL x 3, DMF 15 mL x 3 and DCM 15 mL x 3.

Fmoc deprotection: After each coupling, Fmoc-group deprotection is performed by treating the resin with piperidine in DMF a 20% v/v for 10 min, twice. The resin is then washed with DMF 15 mL 5 times and DCM 15 mL 5 times.

Octa(ethyleneglycol) coupling: 1.5 eq Fmoc-PEG_8-OH was preactivated in DCM:DMF 9:1 with 1.5 eq PyBOP and 4.5 eq. DIEA. The mixture was transferred to the syringe, rocked for 15 h and shaken for 5 min every hour during the first three hours. Then, the syringe was filtered and washed with DCM 15 mL x 3, DMF 15 mL x 3 and DCM 15 mL x 3.

4-(Fmoc-amino)butanoic acid and 8-(Fmoc-amino)octanoic acid coupling: 1.5 eq Fmoc protected building block was preactivated in DCM:DMF 9:1 with 1.5 eq PyBOP and 4.5 eq. DIEA. The mixture was transferred to the syringe, rocked for 15 h and shaken for 5 min every hour during the first three hours. Then, the syringe was filtered and washed with DCM 15 mL x 3, DMF 15 mL x 3 and DCM 15 mL x 3.

Fmoc-L-phenylalanine-4'-azobenzene coupling: 1.5 eq Fmoc-L-phenylalanine-4'-azobenzene-OH was preactivated in DCM:DMF 1:1 with PyBOP 1.5 eq and DIEA 4.5 eq. The mixture was transferred to the syringe, rocked for 15 h and agitated for 5 min every hour during the first three hours. Syringes containing the azobenzene compound were covered with aluminium foil to avoid light exposure. Then, the contents of the syringes were filtered and washed with DCM 15 mL x 3, DMF 15 mL x 3 and DCM 15 mL x 3.

o-Nynhydrin test: (Purchased from Anaspec) Just after an aa coupling, a ninhydrin test was used in order to identify primary amines, so to check if the coupling is completed. The protocol can be found in the datasheet of the product (AS-25241):

1. 20-30 beads of resin were added in a test tube.
2. Two drops reagent 1, 2 and 3 were added.
3. The test tube was heated at 120 °C for 3-4 min.

The test has three possible results:

a) Blue (positive): Primary amines detected

b) Brown-red: Secondary amines detected

c) Pale yellow (no change of color, negative): no amines detected

In the cases of positive result, a recoupling of the same amino acid was required. If the result was negative, we proceeded to the Fmoc deprotection step.

Cleavage: The resin was filtered and transferred to a 50 mL vessel and treated with 1% TFA in DCM for two minutes and filtrated and dried by a gentle stream of N_2. The procedure was performed twice. The product was purified by reversed-phase HPLC and dried in vacuo yielding wedge 1 (C4) and wedge 2 (C8).

C4 wedge

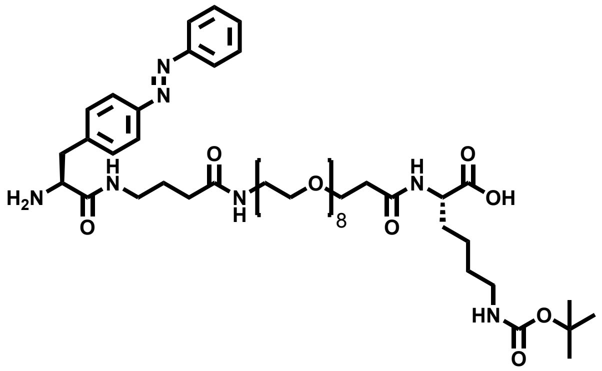

S1. Molecular structure of C4 wedge.

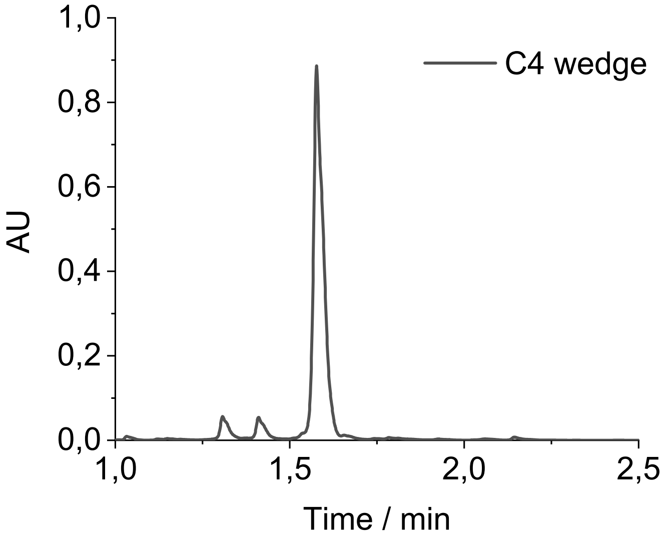

S2. Chromatogram of C4 wedge. Peak at 1.6 min corresponds to *E*-isomer, peak at 1.4 corresponds to the Z-isomer. ESI(+) of wedge **1**. m/z: $[M+H]^{+}$= 1007.0, $[M+2H]^{2+}$= 504.0. Calculated: m/z: $[M+H]^{+}$=1007,21, $[M+2H]^{2+}$= 504.1

C8 wedge

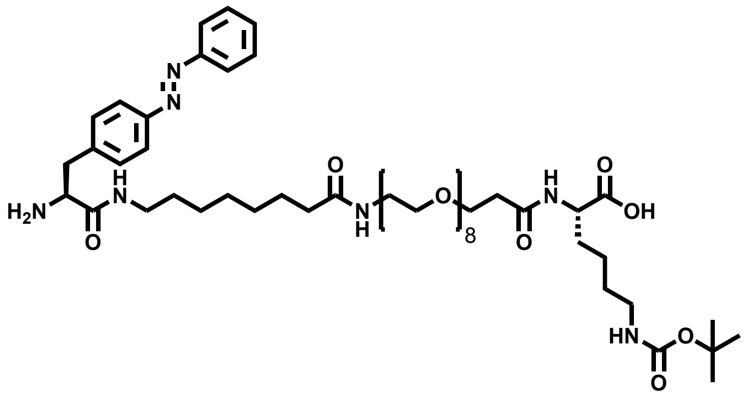

S3. Molecular structure of C8 wedge.

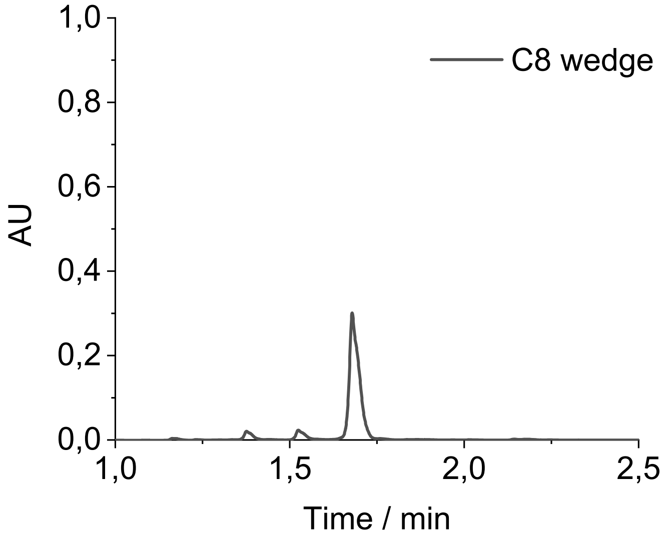

S4**.** Chromatogram of C8 wedge. The peak at 1.55 min corresponds to the *Z*-isomer, the peak at 1.7 min corresponds to the *E*-isomer. ESI(+) of C4. m/z: $[M+H]^{+}$= 1063.1 $[M+2H]^{2+}$= 532.0 m/z Calculated: m/z: $[M+H]^{+}$=1063.3, $[M+2H]^{2+}$= 532.15.

- **Solution synthesis of C3-symmetric monomers**

Monomers C4 and C8 were synthesised by reacting the corresponding wedge with 1,3,5-benzenetricarbonyl trichloride. The wedges (1 eq. 150.0 mg wedge 1, 217.2 mg wedge) and 3 eq of triethylamine were dissolved in $CHCl_3$ (0.5 mL) and cooled to 0 °C. 1,3,5-benzenetricarbonyl trichloride (13 mg for C4 wedge and 15.7 mg for C8 wedge, 0.33 eq) was dissolved in 0.5 mL of $CHCl_3$ and added dropwise to the reaction mixture under stirring at 0 °C. After 15 min at 0 °C, the mixture was stirred another 12 hrs at rt. The mixture was subsequently concentrated *in vacuo* and the resulting oil was dissolved in H_2O:ACN 1:1 and dried *in vacuo*.

Boc cleavage was performed by treating the product with TFA 95% and 5% TIPS (Triisopropyl Silane) for 1 h. The product was then dried by a gentle stream of N_2, dissolved in H_2O:ACN 1:1, purified by reversed-phase HPLC and dried *in vacuo* yielding monomer **C4** and **C8**. (Yield: C4: 23.9% // C8: 33.1%)

C4 monomer

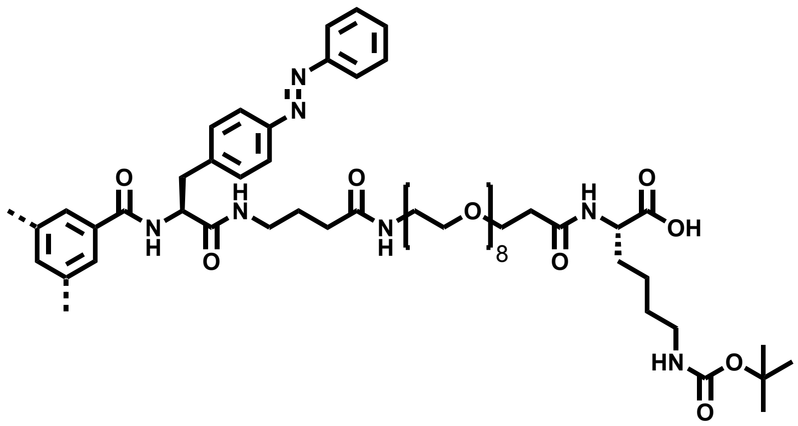

S5. Molecular structure of C4 monomer. MW: 2874.04.

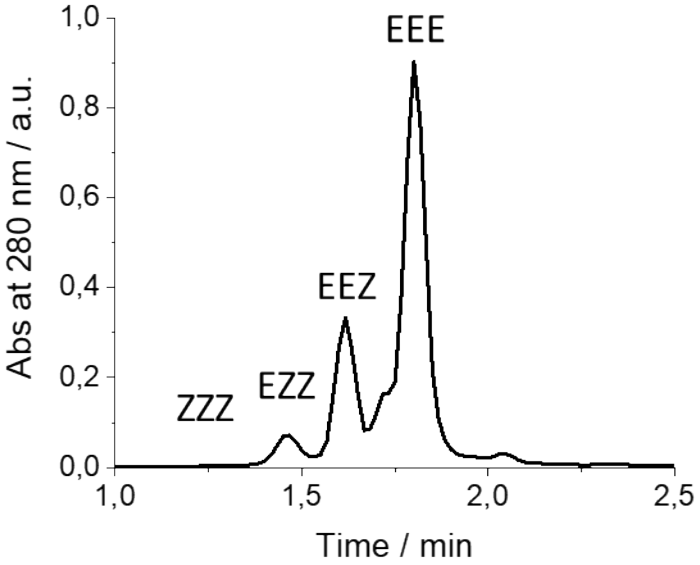

S6. RP-HPLC at 280 nm of C4 monomer. T_R: *EEE* – 1.8 min, *EEZ* – 1.58 min, *EZZ* – 1.45 min.

MS-C4**:** ESI-MS deconvoluted (+): 2873.1. Calculated mass: 2874.4.

MALDI-TOF-MS (α-Cyano-4-hydroxycinnamic acid was used as a matrix)**:** m/z: $[M+H]^+$= 2874.04 (Isotopic distribution can be observed).

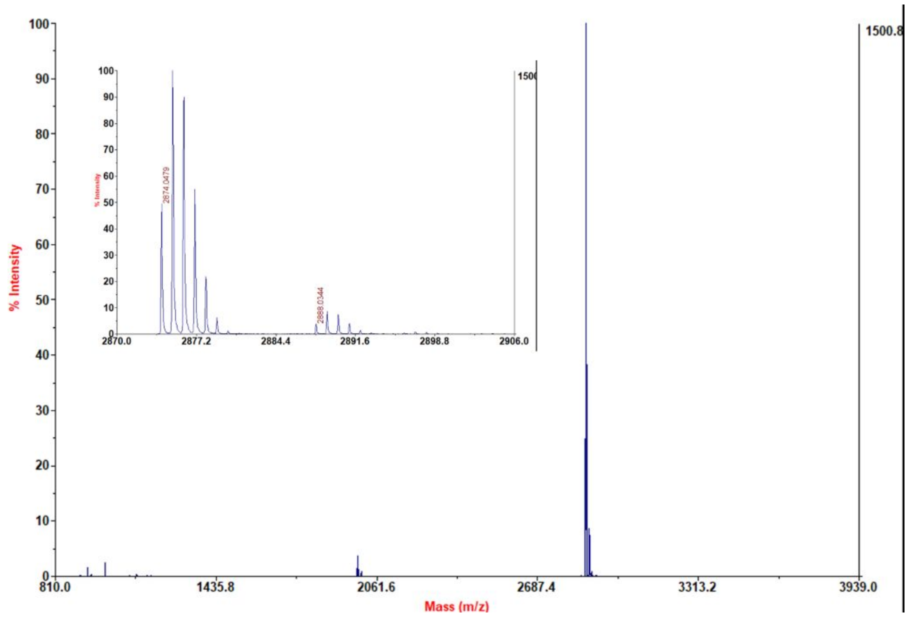

S7. MALDI-TOF spectra of C4 monomer.

^{1}H NMR (400 MHz, DMSO-d_6, E/Z mixture, signals of the main isomer)

δ /ppm = 9.02 (s, 3H), 8.38 (m, 3H), 8.32 (m, 3H), 7.88 (m, 3H), 7.82 (m, 9H), 7.77 (m, 6H), 7.55 (m, 15), 4.78 (m, 3H), 4.04 (m, 3H), 3.57 (m, 12H), 3.48 (m, 87H) partially hidden by water signal), 3.17 (m, 15H), 2.72 (m, 6H), 2.35 (m, 6H), 2.06 (m, 6H), 1.70-1.45 (m, 24H), 1.30 (m, 6H).

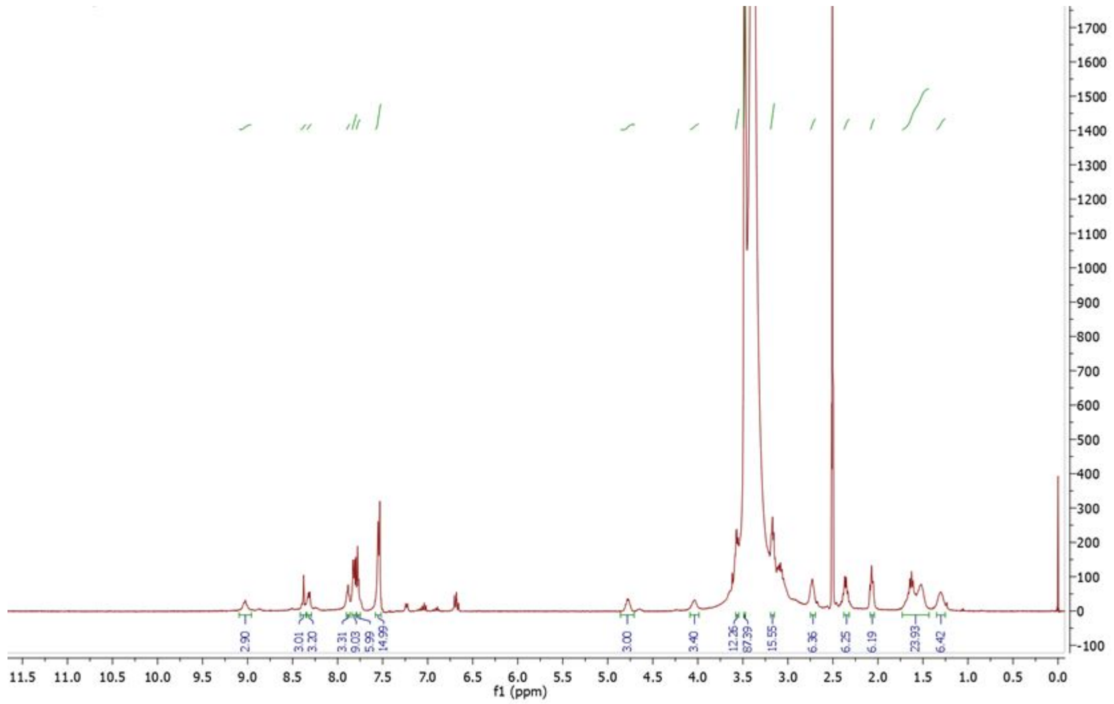

S8. ^{1}H-NMR spectra of C4 monomer.

C8 monomer

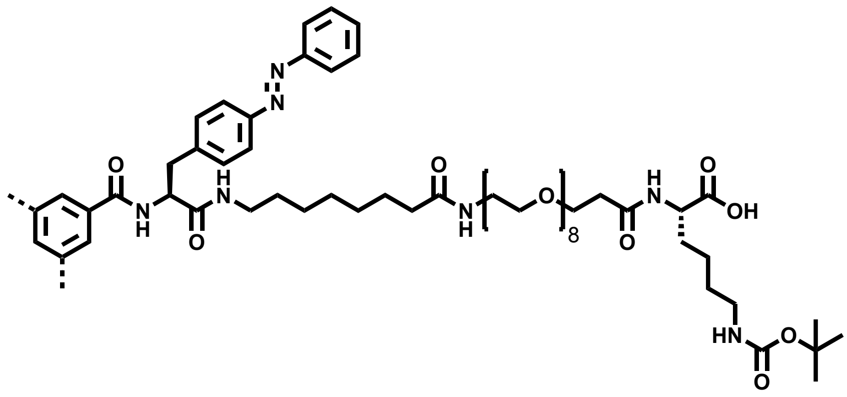

S9. Molecular structure of C8 monomer. MW: 3042.7.

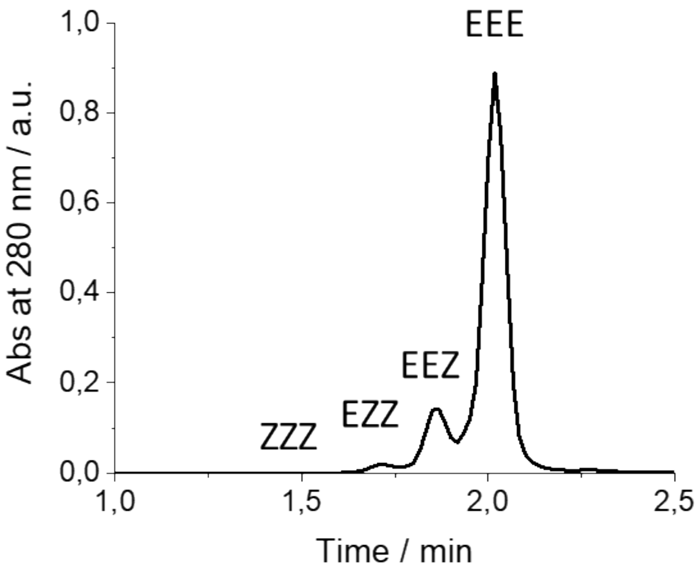

S10. RP-HPLC at 280 nm of C8 monomer. T_R: *EEE* – 2.0 min, *EEZ* – 1.8 min, *EZZ* – 1.7 min.

MS-C8: ESI-MS deconvoluted (+): 3041.9. Calculated mass: 3042.7

MALDI-TOF-MS (α-Cyano-4-hydroxycinnamic acid was used as a matrix)**:** m/z: $[M+H]^+$= 3043.0 (Isotopic distribution can be observed).

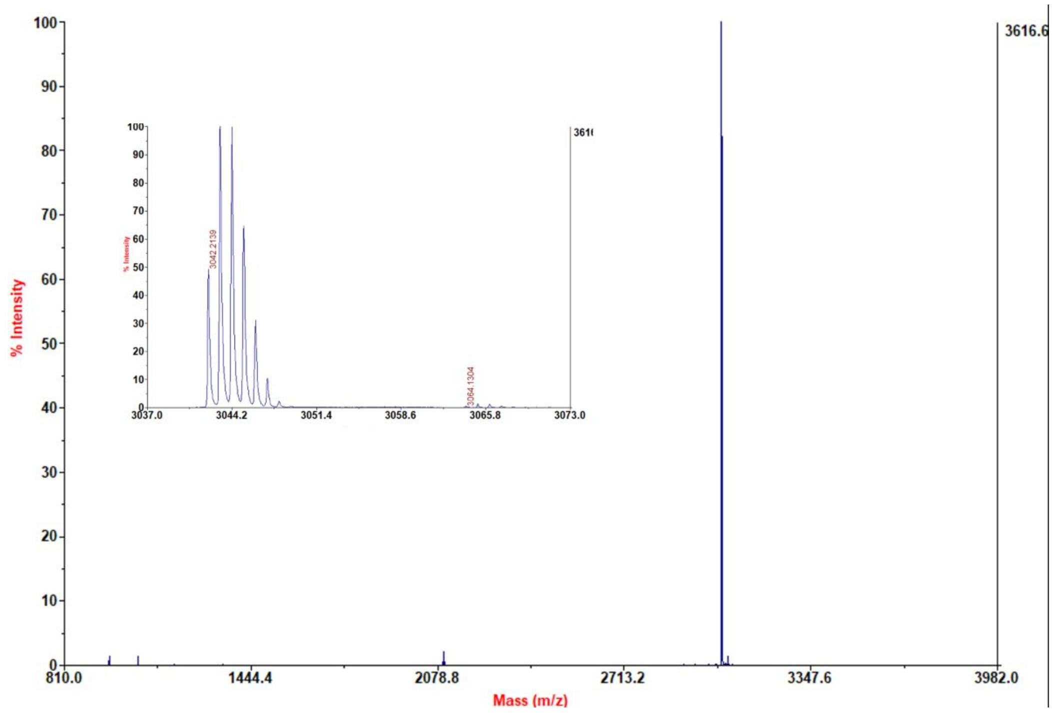

S11. MALDI-TOF spectra of C8 monomer.

^{1}H NMR (400 MHz, DMSO-d_6, E/Z mixture, signals of the main isomer)

δ /ppm = 8.97 (s, 3H), 8.35 (m, 3H), 8.22 (m, 3H), 7.85-7.76 (m, 18H), 7.60-7.51 (m, 15H), 4.79 (m, 3H), 4.04 (m, 3H), 3.54-3.61 (m, 12H), 3.49 (m, 87H) partially hidden by water signal), 3.20-3.12 (m, 15H), 2.73 (m, 6H), 2.37 (m, 6H), 2.01 (m, 6H), 1.67 (m, 3H), 1.52 (m, 9H), 1.42 (m, 6H), 1.32 (m, 12H), 1.18 (m, 24H).

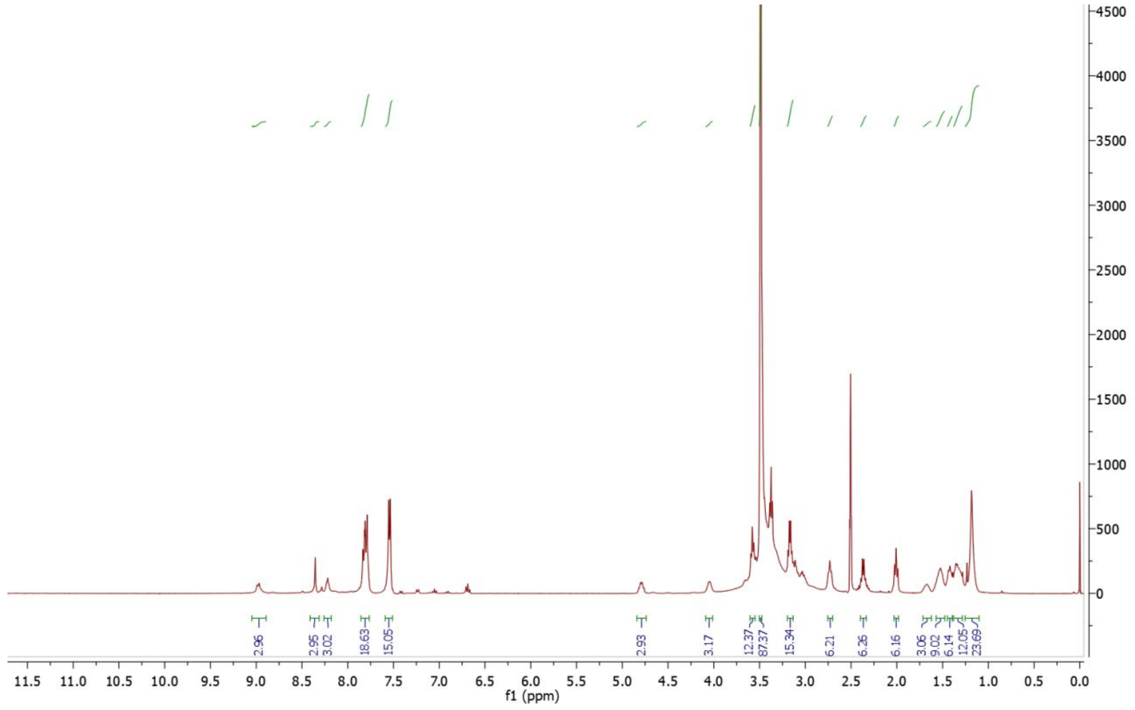

S12. ^{1}H-NMR spectra of C8 monomer.

- **Monomer labelling protocol**

Monomers were labelled by reacting the monomers with sulfo-Cyanine 3 and sulfo-Cyanine 5 NHS ester. C4 and C8 (5 mg , 1 eq) were dissolved in 1mL DMSO at room temperature and put under stirring. 3 eq of TEA were added dropwise. The corresponding NHS-Ester dye, previously dissolved in DMSO (10 mM for sCy3 and 15 mM for sCy5, 1 eq), was added dropwise to the corresponding monomers solution. The reaction was stirred for 20 h under stirring at room temperature. Excess of dye was removed by dialysis. Products were lyophilized, dissolved in H_2O:ACN 1:1 and purified by reversed-phase HPLC and dried *in vacuo* yielding monomer C4-sCy3, C4-sCy5, C8-sCy3 and C8-sCy5. Products were analysed by ESI-MS.

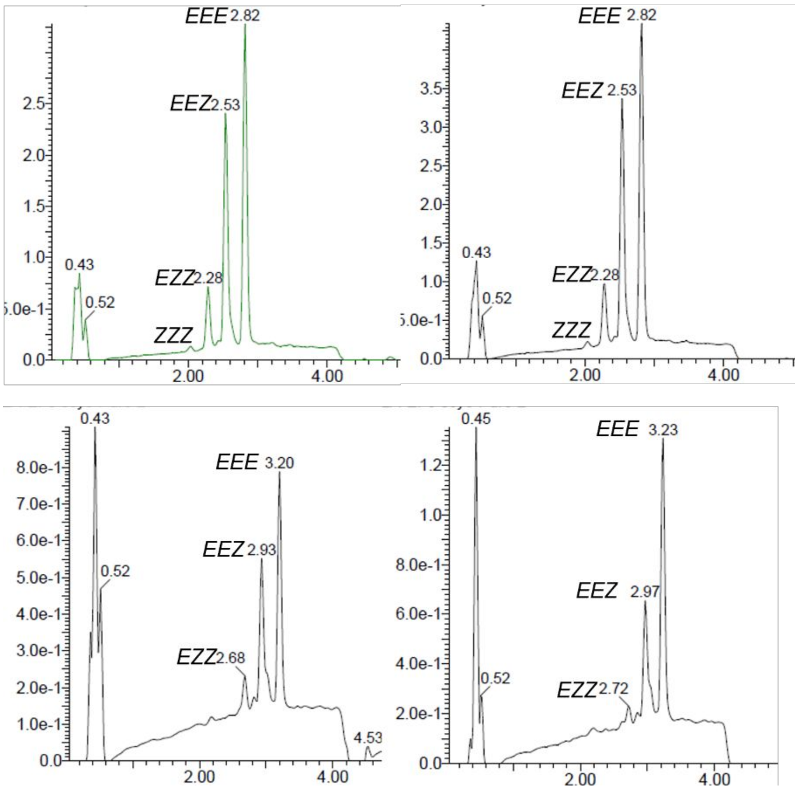

S13. (Top Left) RP-HPLC of C4-sCy3, T_R: *EEE* – 2.8 min, *EEZ* – 2.5 min, *EZZ* – 2.3 min. (Top right) RP-HPLC of C4-sCy5, T_R: *EEE* – 2.8 min, *EEZ* – 2.5 min, *EZZ* – 2.3 min. (Bottom Left) RP-HPLC of C8-sCy3, T_R: *EEE* – 3.2 min, *EEZ* – 2.9 min, *EZZ* – 2.7 min. (Bottom right) RP-HPLC of C8-sCy5, T_R: *EEE* – 2.7 min, *EEZ* – 3.0 min, *EZZ* – 3.2 min.

MS- C4-sCy3: ESI-MS deconvoluted (+): 3485.8. Calculated mass: 3486.1

MS- C4-sCy5**:** ESI-MS deconvoluted (+): 3512.3. Calculated mass: 3512.2.

MS- C8-sCy3**:** ESI-MS deconvoluted (+): 3653.64. Calculated mass: 3654.4

MS- C8-sCy5**:** ESI-MS deconvoluted (+): 3679.97. Calculated mass: 3680.5

3.2 Self-assembly and UV-response

Irradiation set up: Thorlabs M365LP1 was used as UV source at 365 nm, and Thorlabs M455L4 was used as blue source at 455 nm. Irradiations were performed inside a custom-made black box. For the absorbance

experiments and CD experiments, irradiations were performed on the cuvette, at a distance of 10 cm. For the rest, the irradiation was performed on a 96-well plate at a distance of 10 cm.

Sample preparation:

Monomers were dissolved in DMSO at a concentration of 10 mM and labelled monomers were dissolved at 1 mM. If required, labelled and non-labelled monomers were mixed in DMSO achieving the required ratios. Next, concentrated solutions in DMSO were diluted with PBS (pH = 7.4) to obtain a 25 – 100 µM monomer solution (<1% in DMSO), triggering the self-assembly. After, samples were equilibrated for 24h, annealed at 70 °C for 1 h, cooled down to rt and equilibrated for additional 24 h. This procedure was applied to obtain a reproducible state.

In the case of C8, we could asses how the final FRET signal was strongly affected by the sample preparation method. If instead of mixing the labelled monomers in DMSO, we inject them subsequently in water, immediately one after the other, the resulting FRET is below half of the initial value. When sCy3 labelled fibres were mixed with sCy5 fibres, extremely low levels of FRET were observed (S10). This proves the extremely low monomer exchange rates of C8, because once the labelled monomers are assembled, they do not disassemble and reassemble again on another fibre.

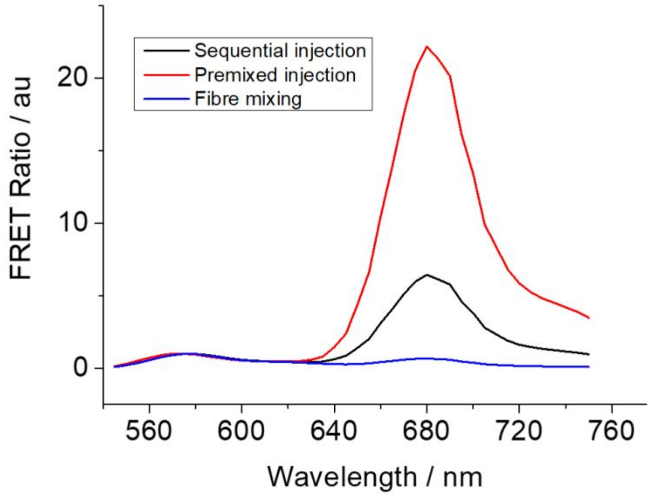

S14. Comparison of FRET spectra of three different preparation methods for labelled fibres. In black, sequential addition of labelled monomers in PBS. In red, single injection in PBS of premixed monomers in DMSO. In blue, mixture of sCy3 labelled fibre with sCy5 labelled fibre. All samples were prepared at a concentration of 25 µM and 10% of labelling.

FRET ratio comparison:

The FRET ratio is calculated by dividing the acceptor fluorescence by the donor fluorescence. This offers a qualitative measurement of the self-assembly and allows to visualize changes in self-assembly. The FRET ratios obtained are 0.5 for C0, 2.5 for C4 and 23 for C8. This trend matches the hydrophobic-hydrophilic balance trend of the molecules, and it is explained by a combination of two parameters: the distance between the dyes and the number of the FRET pair of dyes. That C0 has a low FRET probably indicates that less monomers are aggregates, so more labelled monomers are free. However, C8 has a very high signal, what could be that is has less free monomer than C0 and that dyes are in closer proximity.

Photoisomerization kinetics experiment: Samples of polymers prepared in water and DMSO were irradiate with UV with consecutive irradiations of 5 s to study the kinetics of photoisomerization. C0 and C4 showed similar results in both water and DMSO, being the isomerization very fast (~10 s). However, C8 clearly showed

a decrease in the photoisomerization rate, but only in water. This can be explained by a higher steric hindrance in C8 fibres with respect C0 and C4, because of a different azobenzene packing inside.

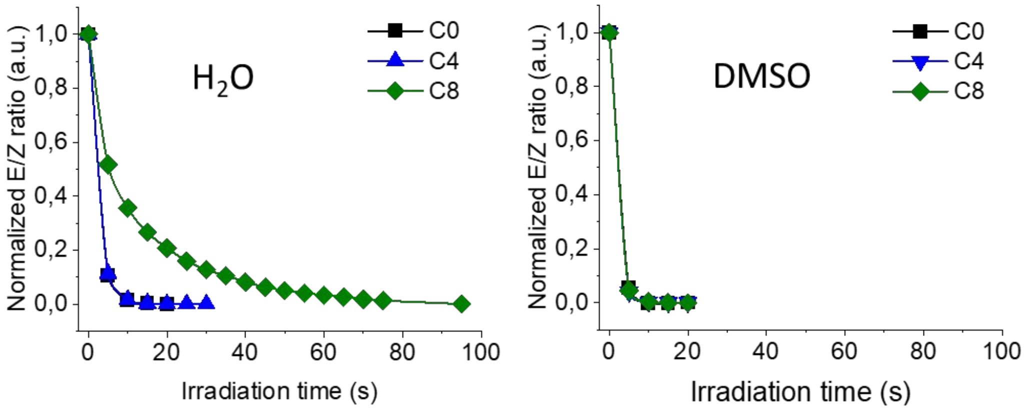

S15. *E-Z* isomerization kinetics, comparison between H_2O (left), PBS and DMSO (right) for monomers at 25 µM. Notice that C0 and C4 curves are overlapped. *E* λ: 330 nm, *Z*-λ: 430 nm.

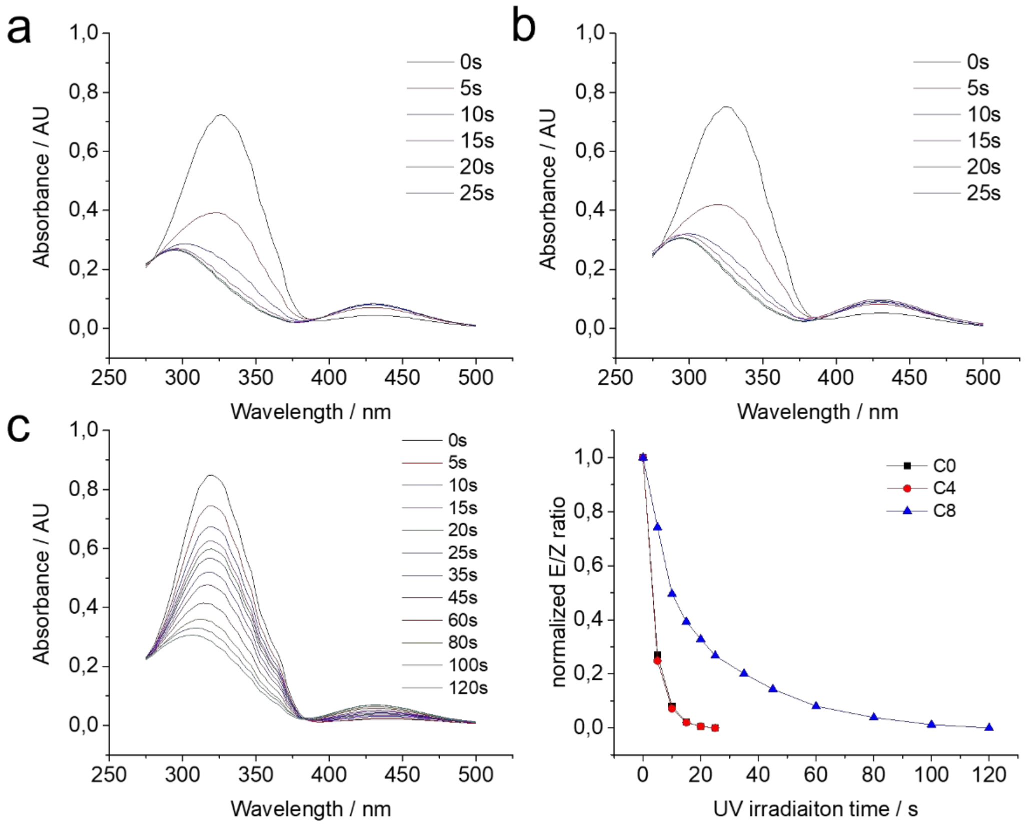

S16. Absorbance spectra of C0 (a), C4 (b) and C8 (c) at 25 uM in PBS. Samples were recorded after short irradiations of 5s with UV light. *E-Z* isomerization kinetics in PBS (d). Notice that C0 and C4 curves are overlapped. *E*-λ: 330 nm, *Z*-λ: 430 nm.

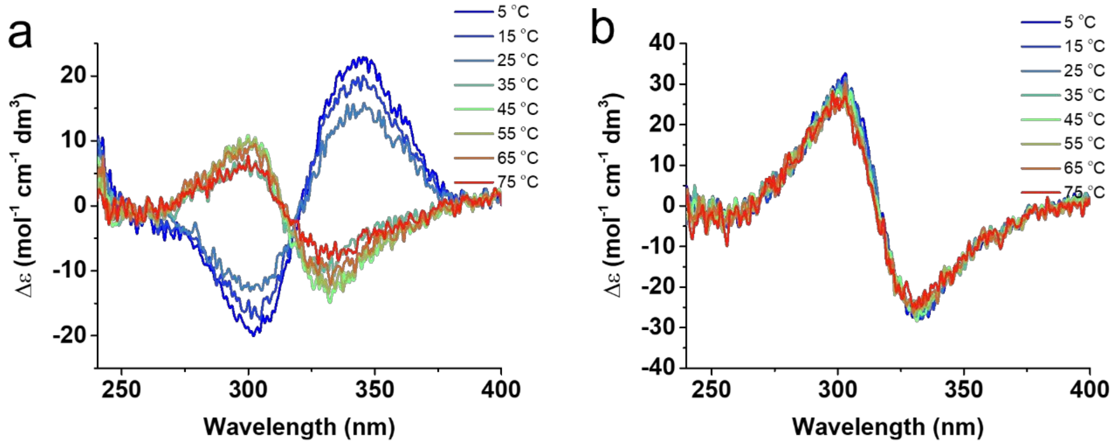

S17. CD spectra at different temperatures of C4 (a) and C8 (b) at 25 µM in PBS.

Photofatigue experiment: Several photoisomerization cycles were performed in order to observe the reversibility of the system. The three monomers showed the same complete reversibility.

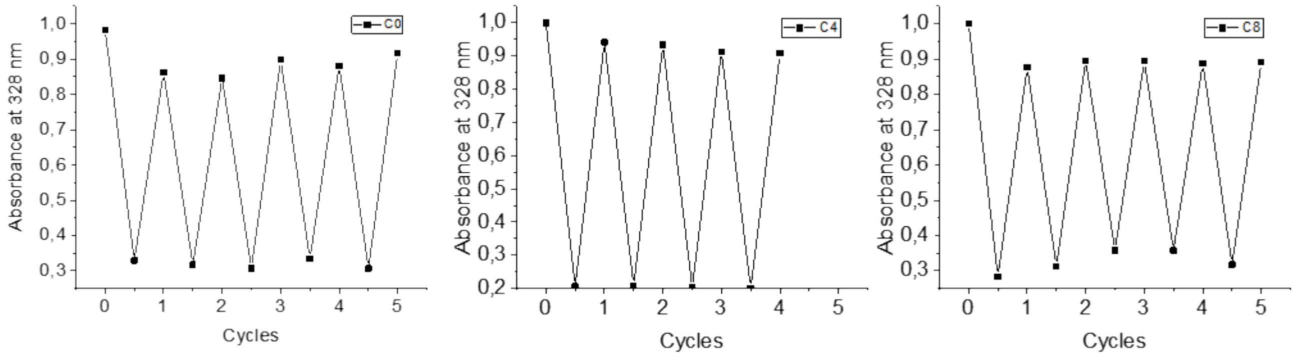

S18. Photofatigue experiment on monomers at 25 µM. Samples were consecutively irradiated at 360nm and 455 nm and measure before each irradiation. No signs of photofatigue were observed for the molecules.

Supramolecular disassembly observed by TEM: Samples from Figure 2 a, were also examined by TEM to provide proof of disassembly triggered by UV irradiation. TEM showed complete disassembly for C0 and C4, however C8 showed a reduction in the fibre length. Interestingly, the fibres could assemble back after irradiation with Vis light, because azobenzenes isomerize back to the trans form. Data for C0 was previously reported.[3]

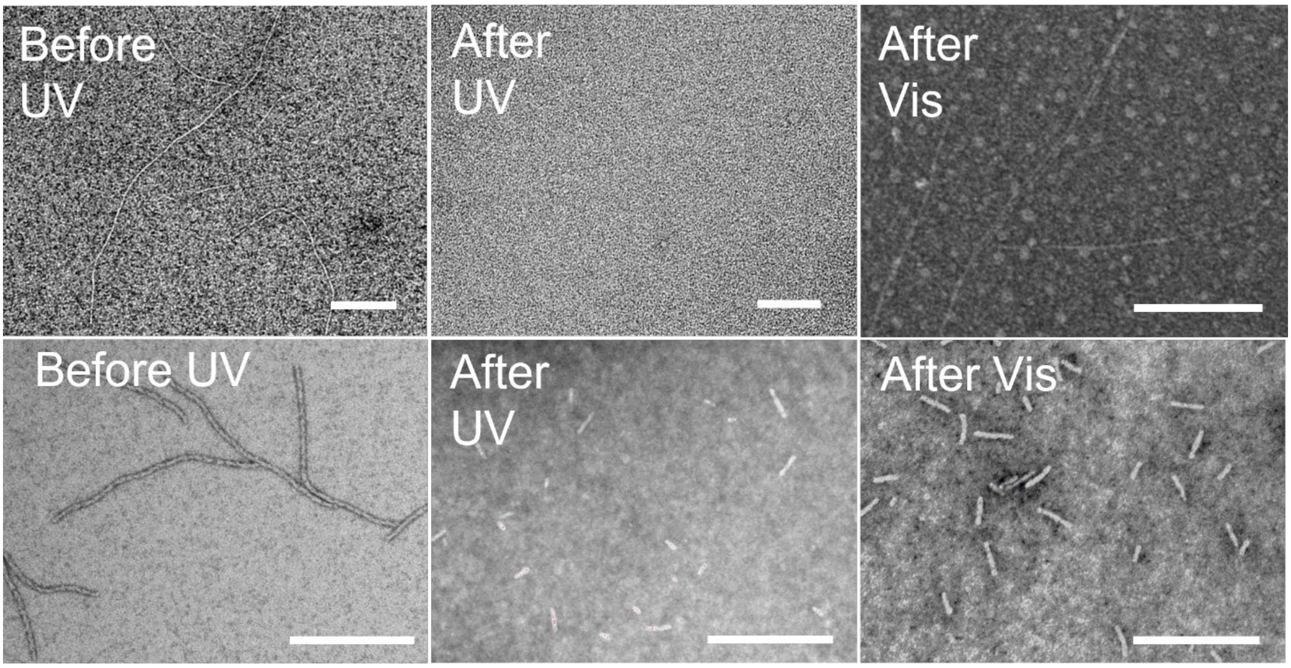

S19. TEM images of C4 (above) and C8 (below) under different irradiations conditions. Scale bar: 200 nm.

Discussion aggregation:

Our model considers only fibres and monomers, however some small species may exist even at extreme dilutions. Achieving a pure free monomeric state is extremely difficult for self-assembled supramolecular polymers. It is possible that very small aggregates of few monomers are present together with the fibres and after UV triggered disassembly of the polymers. However, no conclusive evidence of aggregations after UV irradiation were obtained for C0 and C4. The CD signal from BTA is very noisy due to the presence of DMSO, however, it looks like this signal does not disappear upon irradiation. Also, a low CD signal coming from *Z*-azobenzene appears in the spectrum, however its behaviour has been mostly unpredictable. TEM images have not revealed such small structures before or after irradiation.

As the labelling concentration for each dye is 5% mol, such small aggregates must contain less than 20 monomers to not display FRET signal. We considered that such small aggregates (if they form), would behave very similarly to monomers, and we simplified our model for the sake of clearer explanations.

3.3 Stability in biological-like conditions experiments

Irradiation set up: For CD experiments and HPLC of Figure 3, two LED light sources were used, a Thorlabs M365LP1-C5 was used as UV source at 365 nm (irradiation steps of 1s, 100% at 1000 mA of the LED intensity), and a Thorlabs M455L3-C5 was used as blue source at 455 nm (irradiation steps of 10 s, 100% at 1000 mA of the LED intensity). Irradiation occurred on the bench top at rt, with approximately 15 cm distance between the sample and the LED.

Sample preparation:

- Dilution: samples were prepared initially at 100 µM, and ½ diluted directly in the 96 well plate before recording FRET.
- BSA: BTA samples were prepared at total monomer concentration of 37 µM. Specific amounts of BSA were added to each well and well mixed to reach a final concentration of 25 µM of polymer. The final concentrations of BSA were 0, 10, 20, 30, 40 and 50 mg/ml, that were expressed in terms of BSA excess

(0 to 30 fold relative to total monomer concentration). FRET signal was measured before and after addition of BSA.

- Fibre-BSA FRET: BSA was added at a final concentration of 10 mg/ml (6 fold) and 10% Cy5 labelled. Sample preparation was identical to the previous experiment with BSA.

FRET normalization

In figures 2, 3, S20 b and S21 b, the fluorescence spectra were normalized for the donor peak equaling 1. By this means, the value of the acceptor peak directly represents the FRET ratio, what makes the different FRET graphs easier to compare and changes are easier to visualize.

Assembly % was calculated by normalizing the FRET ratio (acceptor emission peak/donor emission peak), considering the FRET ratio values at 25 µM as 100% assembly, and the FRET ratio obtained after UV irradiation at 25 µM as the 0 % assembly (this point was extracted from Figure 3 C). As C8 was not disassembled completely by UV, FRET ratio after UV from C4 was used as 0. FRET ratios can be observed in figures S20 b and S21 b, by looking at the acceptor peak (sCy5). Then we considered FRET ratio at 25 µM (highest point) as 100% assembly and FRET ratio of figure 3 c as 0 % assembly.

Kinetics of disassembly of supramolecular polymers

The disassembly caused by dilution and BSA addition was also evaluated in time. Figure S14 shows the temporal evolution of the FRET signal in time. In all the cases, for all monomers, the signal showed the major signal decrease from the first measure. Dilution experiment results are in accordance to the behaviour observed for C0 and C4, but apparently not for C8. We expected seeing kinetic effects here due to the high robustness observed. We hypothesise that other effects could interfere, like for example fibre bundling. This could explain a very slow monomer exchange while experimenting some dilution effects. Protein experiments also show a fast equilibration, what indicates a very fast interactions with BSA.

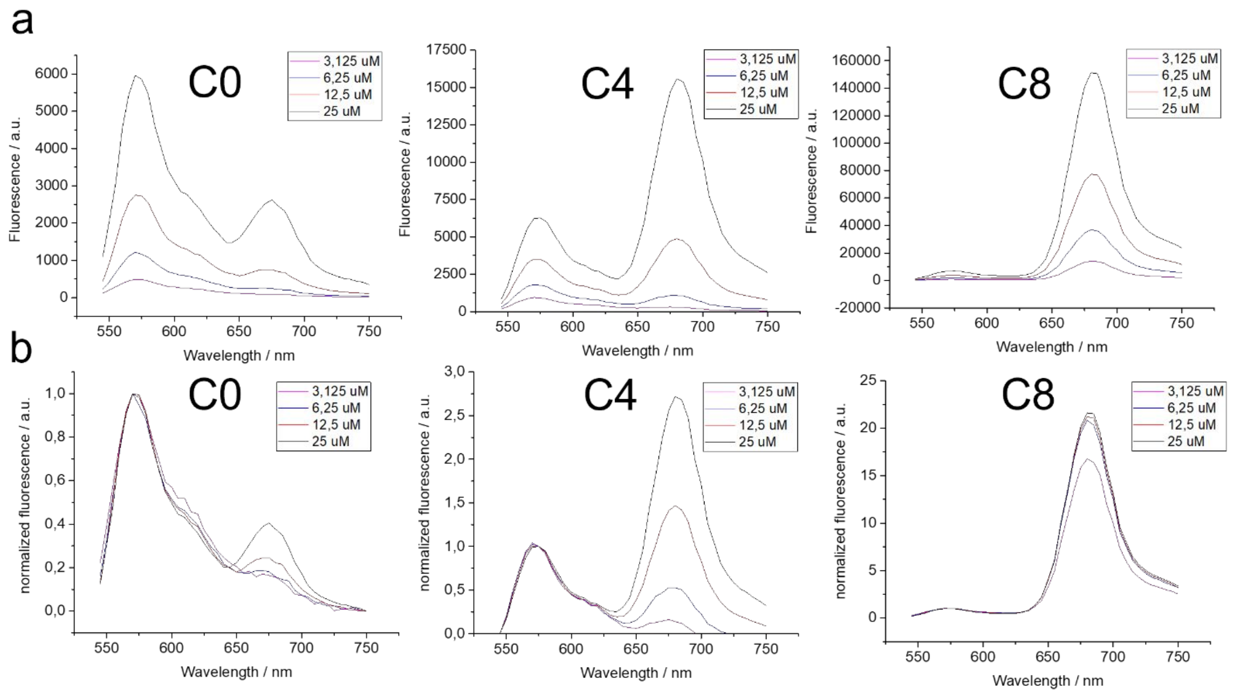

S20. Fluorescence spectra of C0, C4 and C8 at different concentrations after equilibration without normalizing (a) and normalizing (b), where the change in FRET ratio is better appreciated.

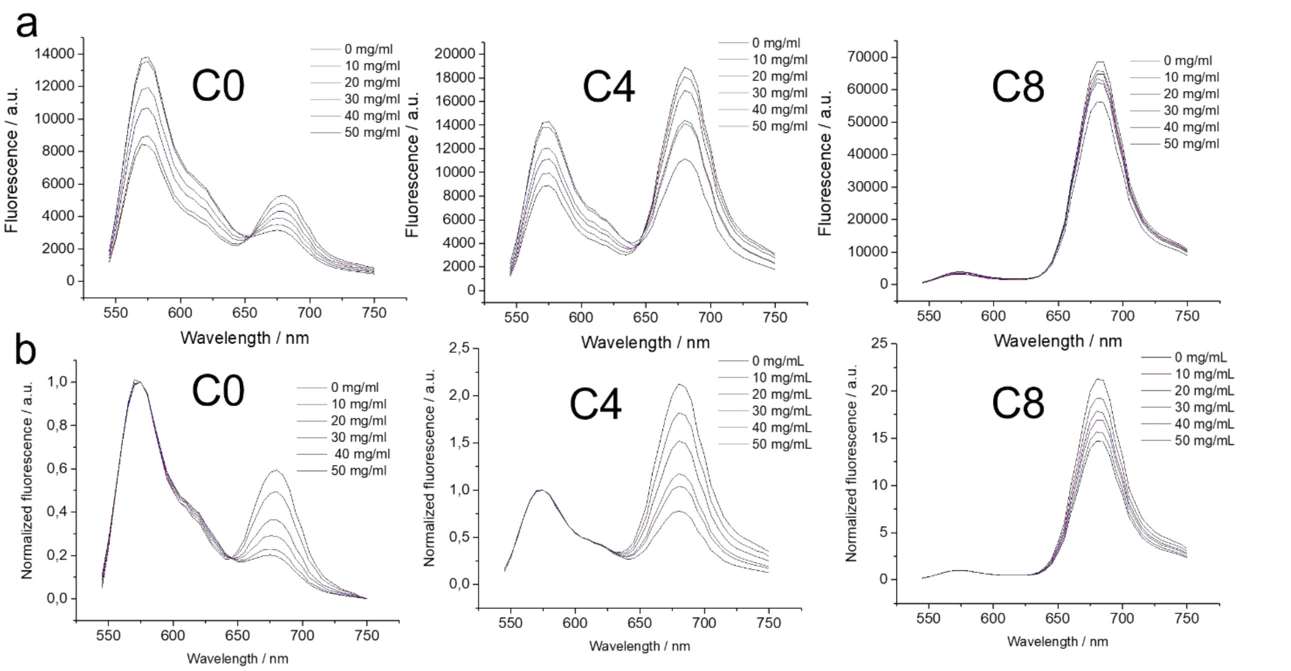

S21. Fluorescence spectra of C0, C4 and C8 at 25 uM at different concentrations of BSA (0 to 50 mg/ml, equivalent to a BSA excess of 6 to 30 fold), without normalizing (a) and normalizing (b).

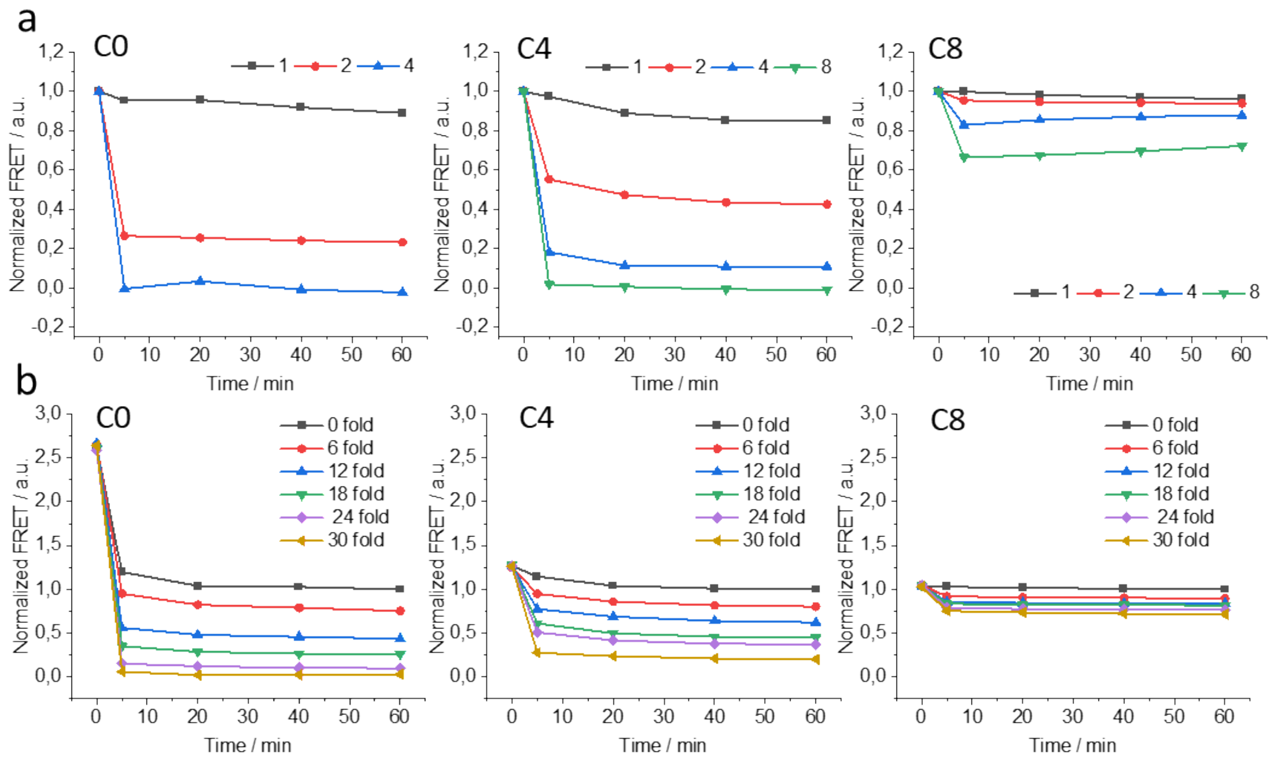

S22. Normalized FRET ratio vs. time after ½ serial dilution (a) and after protein addition (b). Normalized considering FRET ratio at 25 µM as 1, and FRET ratio after UV at 25 µM as 0 (this point was extracted from Figure 3 C). In the case of C8, which complete disassembly was not achieved, FRET ratio of C4 after UV at 25 µM was considered as 0. The initial time point corresponds to the sample at 37 µM, prior the addition of the BSA and dilution to 25 µM.

Photo-response of polymer-BSA FRET

The interaction between BSA and monomers can be tracked also using FRET. In Figure S15 we can observe the relative FRET after irradiating with UV (disassembly) and after Vis (re-assembly). We can observe a slight decrease in the signal of C0 and C4 after UV irradiation, and the recovery after Vis irradiation. We attribute this effect to the loss of fibres and the loss of FRET between fibres and BSA. Interestingly, C8 displays the opposite effect. C8 is more stable and the free monomer concentration is lower, what could be closely related to this effect. However, the exact mechanism is unknown and very challenging to predict.

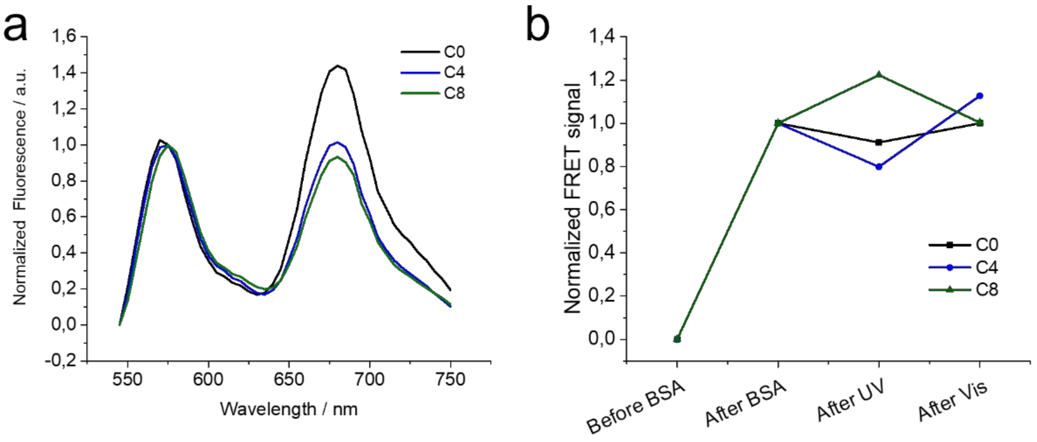

S23. Normalized polymer-BSA FRET signal before and after UV and Vis irradiation.

Bibliography

[1] M. Bose, D. Groff, J. Xie, E. Brustad, and P. G. Schultz, “The Incorporation of a Photoisomerizable Amino Acid into Proteins in E. coli,” *J. Am. Chem. Soc.*, vol. 128, no. 2, pp. 388–389, Jan. 2006.

[2] W. Li, I. Park, S.-K. Kang, and M. Lee, “Smart hydrogels from laterally-grafted peptide assembly,” *Chem. Commun.*, vol. 48, no. 70, pp. 8796–8798, Aug. 2012.

[3] E. Fuentes, M. Gerth, J. A. Berrocal, C. Matera, P. Gorostiza, I. K. Voets, S. Pujals, L. Albertazzi. " An azobenzene-based single-component supramolecular polymer responsive to multiple stimuli in water," J. Am. Chem. Soc., vol. 142, 22, 10069– 10078, May 2020.
